# Genome-Wide Screening of mRNA Expression in Leprosy Patients

**DOI:** 10.3389/fgene.2015.00334

**Published:** 2015-11-20

**Authors:** Andrea de Faria F. Belone, Patrícia S. Rosa, Ana P. F. Trombone, Luciana R. V. Fachin, Cássio C. Guidella, Somei Ura, Jaison A. Barreto, Mabel G. Pinilla, Alex F. de Carvalho, Dirce M. Carraro, Fernando A. Soares, Cleverson T. Soares

**Affiliations:** ^1^Department of Anatomic Pathology, Instituto Lauro de Souza LimaSão Paulo, Brazil; ^2^Department of Anatomic Pathology, A.C. Camargo Cancer CenterSão Paulo, Brazil; ^3^Division of Research and Education, Instituto Lauro de Souza LimaSão Paulo, Brazil; ^4^Department of Health Science, Universidade do Sagrado CoraçãoSão Paulo, Brazil; ^5^Ambulatory of Leprosy, Jardim Guanabara Health CenterRondonópolis, Brazil; ^6^Division of Dermathology, Instituto Lauro de Souza LimaSão Paulo, Brazil; ^7^Laboratory of Genomics and Molecular Biology, CIPE, A.C. Camargo Cancer CenterSao Paulo, Brazil

**Keywords:** leprosy, mRNA, microarray, immunohistochemistry, signaling pathways

## Abstract

Leprosy, an infectious disease caused by *Mycobacterium leprae*, affects millions of people worldwide. However, little is known regarding its molecular pathophysiological mechanisms. In this study, a comprehensive assessment of human mRNA was performed on leprosy skin lesions by using DNA chip microarrays, which included the entire spectrum of the disease along with its reactional states. Sixty-six samples from leprotic lesions (10TT, 10BT, 10BB, 10BL, 4LL, 14R1, and 10R2) and nine skin biopsies from healthy individuals were used as controls (CC) (ages ranged from 06 to 83 years, 48 were male and 29 female). The evaluation identified 1580 differentially expressed mRNAs [Fold Change (*FC*) ≥ 2.0, *p* ≤ 0.05] in diseased lesions vs. healthy controls. Some of these genes were observed in all forms of the disease (CD2, CD27, chit1, FA2H, FAM26F, GZMB, MMP9, SLAMF7, UBD) and others were exclusive to reactional forms (Type “1” reaction: GPNMB, IL1B, MICAL2, FOXQ1; Type “2” reaction: AKR1B10, FAM180B, FOXQ1, NNMT, NR1D1, PTX3, TNFRSF25). In literature, these mRNAs have been associated with numerous pathophysiological processes and signaling pathways and are present in a large number of diseases. The role of these mRNAs maybe studied in the context of developing new diagnostic markers and therapeutic targets for leprosy.

## Introduction

Leprosy is a chronic infectious disease caused by *Mycobacterium leprae*, which affects millions of people worldwide. It is estimated that hundreds of thousands of new cases are diagnosed each year, mostly in India and Brazil, among other countries in South America, Asia, and Africa, in addition to sporadic cases in Europe and North America (World Health Organization, 2014). *M. leprae* is an obligate intracellular parasite with slow replication, a long incubation period, and a small number of genes that control its metabolism (Akama et al., 2009). These characteristics make the host-parasite interaction in leprosy unique, resulting in a chronic spectral long-lasting disease, with various clinical presentations and of great challenge to clinical practice. Ridley and Jopling's classification (R&J) divides leprosy into two polar forms, tuberculoid (TT) and lepromatous (LL), and a borderline group divided into three subgroups: borderline-tuberculoid (BT), borderline-borderline (BB), and borderline-lepromatous (BL), according to clinical, bacilloscopic, and histopathological criteria (Ridley and Jopling, 1966). There may also be two types of reactional episodes, named type 1 reaction (R1) and type 2 reaction (R2), which are sometimes intense and destructive and can occur during disease progression (Hastings et al., 1988). The reactional episodes are the main cause of tissue destruction, particularly nerves, which may result in important sequels and permanent incapacities.

Despite being a long-known disease, there are still major gaps in knowledge about the pathophysiological mechanisms of leprosy (Hastings et al., 1988). Recently, genomic studies have provided a better understanding of pathophysiological mechanisms in several diseases leading to improved diagnosis, prognosis, prevention, and treatment strategies (Pasic et al., 2013). For example, transcriptional profiling of neoplasia, has shown that tumors are molecularly heterogeneous and has helped identify novel genes associated with tumorigenesis and targets with therapeutic, prognostic, or predictive potential (Sethi et al., 2013; Lam et al., 2014). Although similar studies have been conducted in inflammatory and infectious diseases, only few have considered leprosy (Liu et al., 2012; França et al., 2013; Guerreiro et al., 2013; Orlova et al., 2013; Singh et al., 2013; Mehta and Liu, 2014).

The multi-drug therapy (MDT) for leprosy treatment is effective; however, the treatment for reactional episodes is difficult. The drugs most commonly used (corticosteroids and thalidomide), despite effective, are difficult to handle in the clinical practice because they may cause important harmful side effects such as diabetes, Cushing's syndrome, and susceptibility to opportunistic infection, besides the teratogenic effect of thalidomide. Thus, there is a need of new drugs, clinically safer, to be available for leprosy reactional episodes. A comprehensive analysis of human mRNA expression in skin lesions of leprosy patients was carried out in the present study by using DNA chip microarrays which included the entire spectrum of the disease and its reactional forms. The objective was to investigate possible molecular mediators involved in pathophysiological mechanisms of leprosy and to identify biomarkers that could be used as predictive markers of reactions, or contribute to novel therapeutic strategies.

## Materials and methods

### Study design, sample collection, and classification

**Figure 1:**
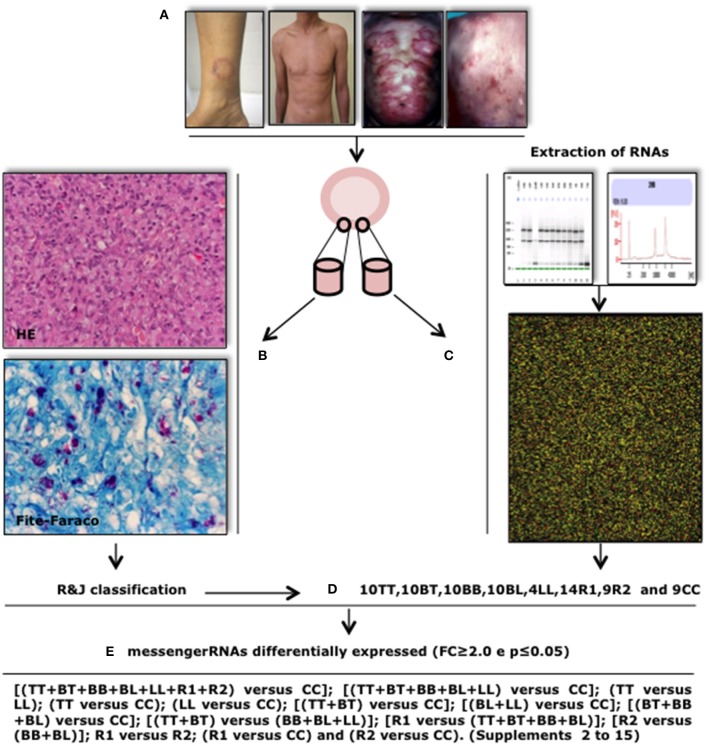
**Study design**. Patients diagnosed with leprosy underwent two punch “5” biopsies on the border of skin lesions **(A)**. One sample was fixed in 10% buffered formalin for routine histological processing and sections were stained with H&E, Fite-Faraco, and immunohistochemistry **(B)**. The second sample was fixed in RNAlater solution for RNA extraction **(C)**. Skin samples from nine healthy subjects were collected and processed in similarly. Samples with good RNA quality were hybridized on microarray plates for identification of differentially expressed mRNAs (*FC* ≥ 2.0, *p* ≤ 0.05) **(C)**. Patients were classified according to Ridley and Jopling's criteria **(D)**. Differentially expressed mRNAs were identified by comparison between the various groups **(E)**.

The sequence of events for this study followed the order below and is detailed in Figure 1. Patients who were treated at the leprosy outpatient clinic of Lauro de Souza Lima Institute-ILSL (Bauru, São Paulo) and the Leprosy Reference Center of Rondonópolis (Rondonópolis, Mato Grosso) were examined by clinical leprologists (CG, SU, and JAB) and submitted to two biopsy procedures, after an informed consent was obtained from the patient. One biopsy was processed for histopathological analysis, bacilloscopy, and immunohistochemistry (IHC) and the other was immediately stored in RNA*later*® solution (Ambion) for further RNA extraction. The non-reactional leprosy patients had never taken any specific leprosy treatment (MDT), or were under any other medication at the time of diagnosis. Besides, they did not present other diseases. In respect to the reactional patients, some developed reactional episodes before leprosy treatment and others presented the reactional episode during or after the treatment. However, none of them was taking any drugs that could interfere with the immune response (corticosteroids or thalidomide). The histopathological exams showed leprosy lesions, with or without reactional pattern, and absence of other concomitant diseases. The control individuals were healthy non-contactants of leprosy patients who were not under any drug treatment. Supplement 1 shows all the data from patients and controls (age, gender, R&J classification, and reactional forms). After clinical, histopathological and bacteriological evaluation, patients were classified according to the R&J criteria including all leprosy forms (TT, BT, BB, BL, LL, R1, and R2) (Ridley and Jopling, 1966; Hastings et al., 1988). Sixty-eight samples from leprotic lesions (10TT, 10BT, 10BB, 10BL, 4LL, 14R1, and 10R2) were selected for analysis. In addition, nine skin biopsies from healthy individuals were used as controls (CC). In order to avoid variations in histological patterns that could interfere with mRNA expression, samples were collected from the trunk and limbs. Scalp, face, palmar, and plantar skin lesions were not included in the study. The study was approved by the Ethics Committees of the AC Camargo Hospital (No. 1535/11) and Lauro de Souza Lima Institute-ILSL (No.03/2011).

### RNA extraction and analysis of quality and integrity

Skin biopsies stored in RNA*later*® were individually cut with a scalpel and transferred to a tube with ceramic beads (CK28, Bertin Technologies). QIAzol reagent (700 μl, Qiagen) was added to samples, followed by processing (homogenization and lysis) in the Precellys24 (Bertin Technologies) apparatus (1 pulse of 10 s and incubation at 4°C for 5 min; cycle was repeated 3 times). Total RNA (including miRNA) was extracted according to the manufacturer's specifications (miRNeasy Mini Kit, Qiagen) by using the QIAcube apparatus (Qiagen). RNA was recovered in 30 μl of RNase-free water and quantified by using the NanoDrop 2000 instrument (Thermo Scientific). RNA integrity was evaluated by using the 2100 Bioanalyzer electrophoresis apparatus (GE Healthcare Bio-Sciences). Samples with low quality or insufficient RNA were excluded from analysis.

### Hybridization of transcripts in the microarray platform

The 8X60K cDNA microarray G4858A platform used in this study (GE Healthcare Bio-Sciences) contained 60,000 probes of 60 base pairs in length representing the entire human genome. Total RNA was reverse transcribed, amplified, and labeled with the fluorophores by using the Low Input AmpLabeling Kit (Agilent Technologies, catalog number 5190-2306) following the protocols recommended by the manufacturer (two-color, microarray-based gene expression analysis; protocol: version 6.5, May 2010, Agilent Technologies).

A competitive hybridization was conducted by using 200 ng of total RNA from the samples marked with Cy5 and 200 ng of reference RNA (pooled RNA from cell lines) labeled with Cy3. Thus, the fluorescence values obtained showed relative levels of expression of each transcript in the test sample compared to the reference sample. Use of the reference sample allowed comparison across independent experiments (Novoradovskaya et al., 2004).

The oligoarrays were hybridized with fluorescent targets for 17 h at 65°C in a hybridization oven, by using the Hi-RPM hybridization buffer (Agilent Technologies). After hybridization, microchips were washed to eliminate non-specific binding and background signals, according to the manufacturer recommendations. The arrays were then scanned by using the Agilent Bundle Scanner (Agilent Technologies) at 3 μm resolution.

### Analysis of microarray results

The microarray data were analyzed by using the Gene Spring GX version 12.1 software (Agilent Technologies). Initially the Control Type, Probe Name, Signal, and Feature data were imported from Feature Extraction program (version 10.7.1, Agilent Technologies). Data was normalized by log transformation and baseline transformation.

Once normalized, the data was filtered to eliminate the spots with low signal, background, or saturation of hybridization. Quality control of the data was conducted by principal components analysis (PCA). The data was then filtered to select only probes with evidence of expression in the samples.

The search for differentially expressed genes was performed by comparison between all the groups. Thus, expression of the control group was compared to all disease groups [(TT + BT + BB + BL + LL + R1 + R2) vs. CC], all forms of the disease [(TT, BT, BB, BL, LL) vs. CC], all intermediate forms [(BT + BB + BL) vs. CC], polar side [(TT + BT) vs. CC and (BL + LL) vs. CC], and polar forms (TT vs. CC and LL vs. CC), and the reactional forms (R1 vs. CC and R2 vs. CC). In leprosy lesions, the polar side forms were compared among them [(TT + BT) vs. (BL + LL)], tuberculoid forms and non-tuberculoid forms [(TT + BT) vs. (BB + BL + LL)] and the polar forms among them (TT vs. LL). Similarly, the reactional forms were compared to their respective clinical forms [(TT + BT + BB + BL vs. R1) and (BL + LL vs. R2)], and finally, the reactional forms were compared with each other (R1 vs. R2). The *t*-test with the Bonferroni FWER correction were used for statistical analysis, and differentially expressed genes were considered as those with *FC* ≥ 2.0 and *p* ≤ 0.05. Differentially expressed genes were grouped by a hierarchical cluster unsupervised analysis.

### Validation of differentially expressed mRNAs by using quantitative RT-PCR

Differentially expressed genes identified in the cDNA microarray assay were validated by quantitative RT-PCR assay by using a customized detection system, SyBr Green platform PCR Array® (Qiagen), with 88 target genes, 5 housekeeping genes, and 3 internal control genes. Validation was performed for 24 samples, three of each group (3TT, 3BT, 3BB, 3BL, 3LL, 3R1, 3R2, and 3 controls) including both samples used in the microarray assay and new samples.

The RNA samples were treated with DNase (RNase-Free DNase Set kit, Qiagen) to eliminate residual genomic DNA. Subsequently, RNA was purified and concentrated with the RNeasy MinElute Cleanup Kit (Qiagen) according the manufacturer's specification. The RT^2^ First Strand Kit (Qiagen) was used for cDNA synthesis. The efficiency of the reverse transcription reaction was assessed by using a RT-PCR reaction for GAPDH.

The ABI ViiA 7 instrument was used for real-time PCR reactions (Applied Biosystems) following the RT2 Profiler PCR Arrays Kit (Qiagen) protocol in combination with RT2 SYBR Green Master mix (Qiagen).

The mRNAs were selected after expression analysis in all comparisons. The authors chose the most up and down regulated present in all groups, and therefore, those who were related to the disease. Others were selected because they were differentially expressed in particular forms of the disease or reaction.

### Analysis of quantitative RT-PCR assays

The data quality was analyzed by using the SDS 2.3 software (Applied Biosystems). The threshold or threshold fluorescence intensity were adjusted in the exponential phase chart by using either the amplification curves or amplification plots, and the Ct values (cycle threshold) were selected for each reaction. Results were considered acceptable if HGDC (human genomic DNA control) samples showed amplification after cycle 35 and if standard deviation of duplicates was *Ct* < 0.5.

Quantitative RT-PCR results were analyzed by using the relative expression values obtained by comparison between the expression level of the target gene and the control or reference gene, internal, or normalizer housekeeping gene.

Relative expression: 2-ct (target genes)/2-ct (housekeeping).

The geNorm software that assesses the stability of the genes and the convenience of using more than one gene and normalize was used to select the appropriate housekeeping genes (Vandesompele et al., 2002). Among the five genes included in the PCR array, only three were used in the calculation the relative expression (GAPDH, HPRT1, and RPLP0).

After calculating the relative gene expression of each sample, the difference in expression between groups or fold change (FC) was calculated. The mean values of normalized relative expression of the groups were compared, and the differentially expressed genes were considered as those with *FC* ≥ 2.0 and *p* ≤ 0.05.

### Selection and validation of mRNAs by IHC

Fifteen of the 86 mRNAs subjected to validation by quantitative RT-PCR, were selected for evaluation of protein expression by immunohistochemistry (IHC). Markers that were commercially available and validated in the literature were chosen after an analysis similar to that used for RT-PCR validation. These included ANGPTL4 (angiopoietin-like4, 11F6C4, mouse, 1:50, Abcam, Cambridge, UK); BAI1 (brain-specific angiogenesis inhibitor 1, polyclonal, rabbit, 1:800, Abcam, Cambridge, UK); BCAT1 (branched chain amino-acid transaminase 1, cytosolic, polyclonal, rabbit, 1:400, Abcam, Cambridge, UK); CD2 (CD2 molecule, AB75, mouse, 1:100, Novocastra, Newcastle, UK); CD27 (CD27 molecule, EPR8569, rabbit, 1:800, Abcam, Cambridge, UK); CD52 (CD52 molecule, HI186, mouse, 1:800, Abcam, Cambridge, UK); EML2 (echinoderm microtubule associated protein like 2, polyclonal, mouse, 1:500, Abcam, Cambridge, UK); FA2H (2-hydroxylase fatty acid, polyclonal, rabbit, 1:500, Abcam, Cambridge, UK); GZMB (granzyme B, GZB01, mouse, 1:100, DBS, Pleasanton, USA); LIPA (lysosomal acid lipase, polyclonal, rabbit, 1:400, Abcam, Cambridge, UK); MMP9 (matrix metallopeptidase 9, EP1254, rabbit, 1:400, Abcam, Cambridge, UK); NCF1 (neutrophil cytosolic factor 1, polyclonal, rabbit, 1:1000, Abcam, Cambridge, UK); PTX3 (pentraxin 3, long, MNB1, rat, 1:400, Abcam, Cambridge, UK); SIGLEC15 (sialic acid binding Ig like lectin-15, polyclonal, rabbit, 1:400, Abcam, Cambridge, UK) and UDB (ubiquitin D EPR4370, rabbit, 1:500, Abcam, Cambridge, UK).

IHC was performed using the indirect method with streptavidin-biotin peroxidase (LSAB, Dako) according to the specifications recommended by the manufacturer. The expression of these markers was analyzed in all common components of the skin and also in cells that composed the granuloma and other inflammatory infiltrates (granulomas, neural branches, vessels, arrector pili muscle, skin appendages, stroma, and epidermis).

### Analysis of signaling pathways and cellular processes

Ingenuity pathway analysis software (IPA) was used to better understand the regulatory mechanisms involved in inducing gene expression changes and their impact on diseases of interest through interactive visual exploration of causality between molecules and disease, function, or phenotypes.

### Microarray data accession number

The microarray data set has been submitted to the Gene Expression Omnibus database at NCBI (http://www.ncbi.nlm.nih.gov/geo/) and assigned accession number GSE74481.

## Results

### Differentially expressed mRNAs in the disease and reactional states

Between disease and healthy controls [(TT + BT + BB + BL + LL + R1 + R2) vs. CC], 1580 mRNAs (756up-regulated and 824 down-regulated) were differentially expressed (Supplement 2). In clinical forms vs. CC [(TT + BT + BB + BL + LL) vs. CC], 1145 mRNAs (628 up-regulated and 517 down-regulated) were differentially expressed (Supplement 3 and Table 1). Among the polar forms (TT vs. LL), 33 mRNAs (01 up-regulated and 32 down-regulated) were differentially expressed (Supplement 4). In Supplements 5–10 are detailed all mRNAs differentially expressed comparing TT vs. CC, LL vs. CC, T side (TT + BT) vs. CC, L side (BL + LL) vs. CC, borderline forms (BT + BB + BL) vs. CC, and tuberculoid forms (TT + BT) vs. non-tuberculoid (BB + BL + LL), respectively.

**Table 1:** **The 10 most up or down-regulated mRNAs differentially expressed in the microarray and RT-PCR values for validated mRNAs in clinical forms (TT+BT+BB+BL+LL) vs. CC, *FC* ≥ |2| and *p* ≤ 0.05**.

**Gene symbol**	**Regulation**	**FC microarray**	**FC RT-PCR**
IGLL5	Up	75.7	438.8
UBD	Up	57.0	80.5
GZMA	Up	40.2	
MMP9	Up	35.3	59.7
SLAMF7	Up	32.4	166.2
CHIT1	Up	27.1	985.3
CD2	Up	25.1	52.8
CD3G	Up	21.4	31.4
FAM26F	Up	20.8	25.5
ITGAL	Up	20.1	99.6
PON3	Down	−4.0	−3.3
SLC15A1	Down	−4.1	−2.4
FGFBP1	Down	−4.3	−2.5
NNAT	Down	−4.6	
KRT6C	Down	−4.8	−2.2
FASN	Down	−4.8	−3.5
AADACL3	Down	−5.3	−7.6
KLK6	Down	−7.5	−4.5
FA2H	Down	−8.8	−3.1
FADS2	Down	−9.2	−4.1

In the type “1” reaction vs. its respective clinical forms [R1 vs. (TT + BT + BB + BL)], 55 mRNAs (17 up-regulated and 38 down-regulated) were differentially expressed (Supplement 11 and Table 2). In type “2” reaction vs. its respective clinical forms [R2 vs. (BL + LL)], 25 mRNAs (15 up-regulated and 10 down-regulated) were differentially expressed (Supplement 12 and Table 3). When comparing the reactions (R1 vs. R2), 45 mRNAs (33 up-regulated and 12 down-regulated) were differentially expressed (Supplement 13 and Table 4). In Supplements 14 and 15 are detailed all mRNAs differentially expressed comparing R1 vs. CC and R2 vs. CC, respectively.

**Table 2:** **The 10 most up or down regulated mRNAs differentially expressed in the microarray and RT-PCR values for validated mRNAs in R1 vs. R1 related clinical forms [R1 vs. (TT+BT+BB+BL)], *FC* ≥ |2| and *p* ≤ 0.05**.

**Gene symbol**	**Regulation**	**FC microarray**	**FC RT-PCR**
ADAMTS4	Up	2.9	3.0
NCF1	Up	2.6	
BCAT1	Up	2.6	
RASSF4	Up	2.3	
SLC16A3	Up	2.2	
C17orf96	Up	2.2	
ITGB2	Up	2.2	
PLAUR	Up	2.2	
KCNE3	Up	2.1	
ALOX5	Up	2.1	
GRHL2	Down	−2.7	
FGFR3	Down	−2.7	
CLDN1	Down	−2.6	
NMU	Down	−2.5	
CYP2J2	Down	−2.4	
PPP1R14C	Down	−2.4	
KLF5	Down	−2.3	
HSD11B2	Down	−2.3	
SCEL	Down	−2.2	
TPD52L1	Down	−2.2	

**Table 3:** **The 10 most up or down-regulated mRNAs differentially expressed in the microarray and RT-PCR values for validated mRNAs in R2 vs. R2 related clinical forms [R2 vs. (BL+LL)], *FC* ≥ |2| and *p* ≤ 0.05**.

**Gene symbol**	**Regulation**	**FC microarray**	**FC RT-PCR**
PTX3	Up	47.6	77.6
KRT6C	Up	19.5	111.5
KRT6A	Up	18.7	
AKR1B10	Up	17.6	17.7
IL1B	Up	10.4	34.1
ANGPTL4	Up	7.3	
ADAMTS4	Up	6.9	14.2
NNMT	Up	4.9	2.5
GJB2	Up	4.8	
MT2A	Up	4.5	4.3
NR1D1	Down	−6.7	−14.7
MMP9	Down	−4.3	
TOX2	Down	−3.9	−10.6
TEF	Down	−3.7	−6.6
EPHB6	Down	−3.3	
BAI1	Down	−2.9	−6.0
RAB37	Down	−2.9	
MFSD2A	Down	−2.8	
TNNI2	Down	−2.8	
EEF2K	Down	−2.4	

**Table 4:** **The 10 most up or down-regulated mRNAs differentially expressed in the microarray and RT-PCR values for validated mRNAs in R1 vs. R2, *FC* ≥ |2| and *p* ≤ 0.05**.

**Gene symbol**	**Regulation**	**FC microarray**	**FC RT-PCR**
SIGLEC15	Up	6.6	4.7
MMP9	Up	6.6	13.9
FAM180B	Up	4.5	
SARDH	Up	4.4	5.7
TOX2	Up	4.1	8.0
CHIT1	Up	3.7	15.4
RAB37	Up	3.4	
CD2	Up	3.2	3.0
CXorf65	Up	3.0	
ITGAL	Up	2.9	3.3
PTX3	Down	−27.1	−21.4
AKR1B10	Down	−11.1	−5.9
ANGPTL4	Down	−6.3	
GJB2	Down	−4.2	
NNMT	Down	−3.7	
CD300E	Down	−3.7	−12.2
FAM83D	Down	−3.3	
MDFI	Down	−3.3	
MPZL2	Down	−2.9	
B3GNT5	Down	−2.2	

Biofunction analysis of mRNA expression revealed a large number of canonical pathways involved with the clinical forms and leprosy reactions. Comparison of clinical forms of the disease with controls [(TT + BT + BB + BL + LL) vs. CC] demonstrated that the 8 canonical pathways involved have 50% or more dysregulated genes, related to Antigen Presentation Pathway, Cytotoxic T Lymphocyte-mediated Apoptosis of Target Cells, and B Cell Development. Supplement 16 contains the 8 canonical pathways and the dysregulated genes involved in those pathways.

In a comparison of the type 1 reaction with its respective clinical forms [R1 vs. (TT + BT + BB + BL)], 69 canonical pathways were identified, which play roles in Leukocyte Extravasation Signaling, MSP-RON Signaling Pathway, Glioma Invasiveness Signaling and IL-17 Signaling. Supplement 17 contains all 69 canonical pathways and their respective mRNAs.

When comparing the type 2 reactional state with the respective clinical forms [R2 vs. (BL + LL)], 26 canonical pathways were identified which mainly function in angiogenesis, inhibition of matrix metalloproteases, granulocyte adhesion, diapedesis, and leukocyte extravasation signaling. Supplement 18 contains all 26 canonical pathways and their respective mRNAs.

### RT-PCR validation of differentially expressed mRNAs in the disease and reactional states

Of 77 samples used in the DNA chip microarray, 24 were subjected to RT-PCR validation. This subgroup was represented by three samples of each clinical form including leprosy reactional states and controls. Supplement 19 contains a table with the values of the 87 mRNA samples submitted to validation by RT-PCR, comparing disease, clinical forms, reactional states with control, reactional states with their respective clinical forms, and between reactions. In all, 69 (79.2%) mRNAs were validated (Supplement 19 and Figure 4).

A heterogeneous expression profile was observed. The vast majority of these mRNAs show differential expression in the disease and the reactional states, with either higher or lower expression values between them (CD2, CD27, chit1, FA2H, FAM26F, GZMB, MMP9, SLAMF7, UBD). Only a few, however, are expressed in specific segments of the disease (type “1” reaction: ADAMTS4, ALOX15B, FGFBP1, FOXQ1, GAL, GPNMB, IL1B, LGMN, MICAL2, TMEM91; Type “2"reaction: ADAMTS4, AKR1B10, ALOX15B, FAM180B, FOXQ1, GAL, LGMN, NNMT, NR1D1, PTX3, TNFRSF25). Rare mRNAs showed inversion in expression when reactional states were compared to the disease, meaning that they were down-regulated in the disease and up-regulated in the reactional forms or vice versa (GJB2 and KRT6C).

### IHC validation of differentially expressed genes in the disease

**Figure 2:**
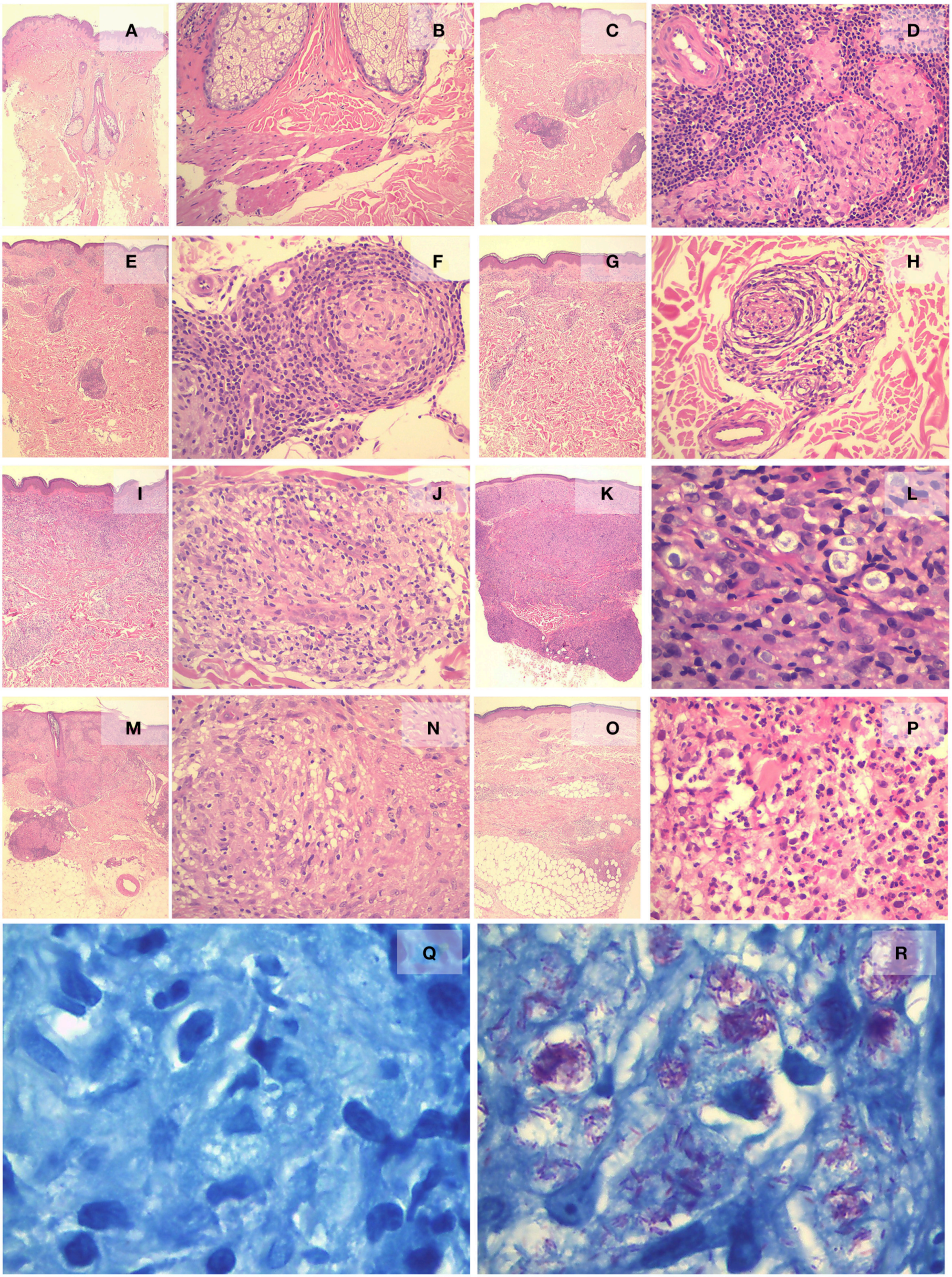
**Histological sections of skin biopsies stained with H&E and Faraco-Fite**. CC showing skin and subcutaneous tissue without inflammatory process (**A**, H&E x2 and **B**, H&E x20). In TT, there are tuberculoid granulomas with epithelioid macrophages in the center and lymphocytes at the periphery (**C**, H&E x2 and **D**, H&E x20). In BT, the granulomas are similar to those of TT (**E**, H&E x2), in which granuloma involves a nerve branch (**F**, H&E x20). In BB, the inflammatory infiltrate is less intense (**G**, H&E x2) and granulomas are less defined with lymphocytes and macrophages involving neural branches (**H**, H&E x20). The inflammatory infiltrate in BL is similar to BB, but more extensive with a larger number of macrophages, lymphocytes and plasma cells (**I**, H&E x2 and **J**, H&E x20). In LL, the infiltrates are dense, occupying all segments of the skin and subcutaneous tissue (**K**, H&E x2), and they consist of multivacuolated macrophages with rare permeating lymphocytes (**L**, H&Ex40). In R1, granulomas are defined with little inflammatory infiltrate extending adjacent to the interstitium (**M**, H&E x2), and the pre-existing granulomas are permeated by young macrophages and lymphocytes and occasionally exhibit necrosis (**N**, H&E x40). In R2, the inflammatory infiltrates involve all layers of the dermis with clusters of neutrophils forming micro abscesses (**O**, H&E x2 and **P**, H&E x40). Bacilloscopy 0+ in TT sample (**Q**, Fite-Faraco x100) and 6+ in LL sample (**R**, Faraco-Fite x100).

**Figure 3:**
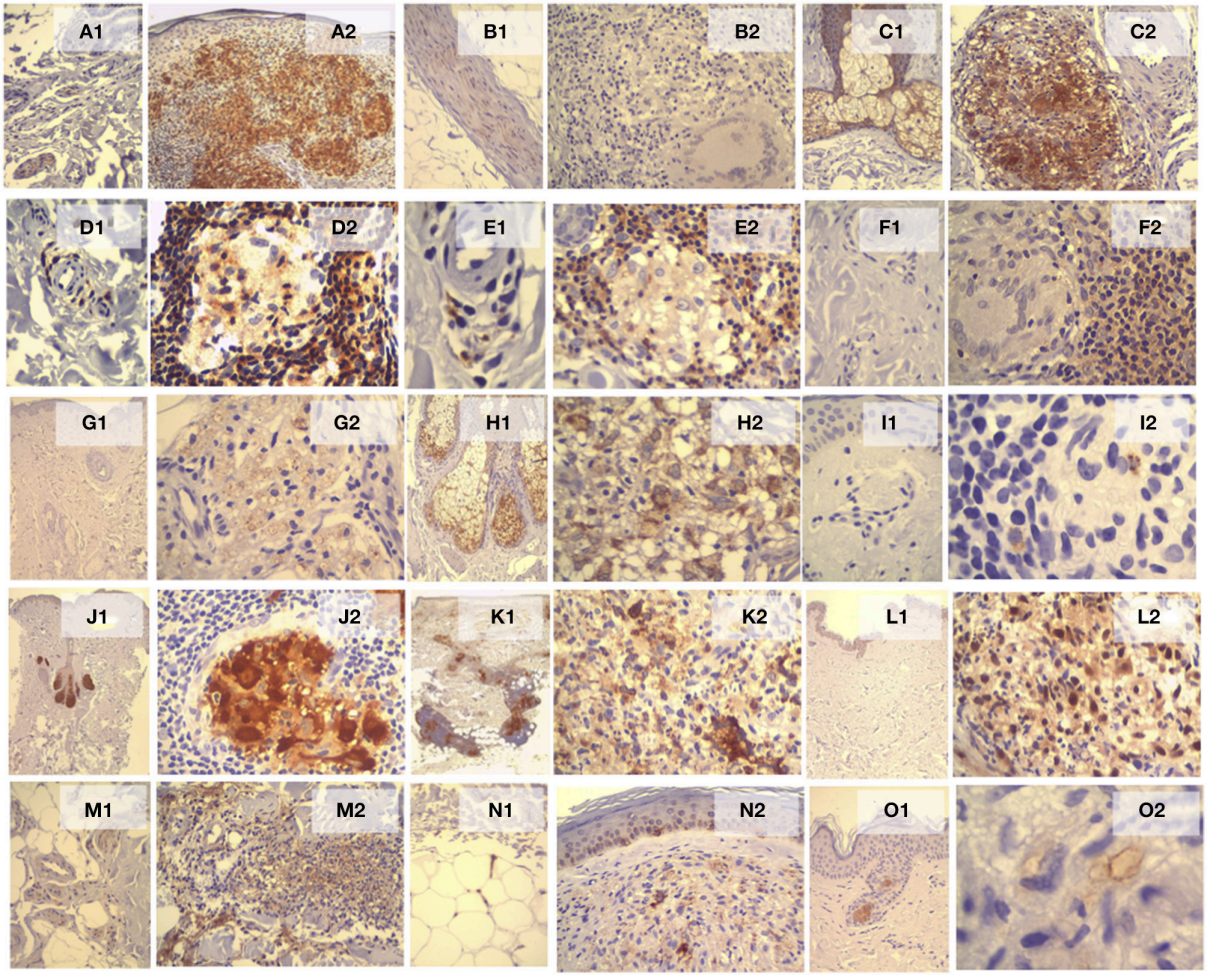
**Histological sections of skin biopsies stained by IHC**. ANGPTL4 expression in neural branches (Schwann cell) and luminal in sweat glands of CC (**A1**, IHC x20); strong expression in macrophages in TT granulomas (**A2**, IHC x10). Weak expression of BAI1 in neural branches of CC (**B1**, IHC x20) and absence of granulomas R1 (**B2**, IHC x20). Weak expression of BCAT1 in sebaceous gland CC (**C1**, IHC x10) and strong in macrophages of R1 granulomas (**C2**, IHC x20). CD2 positive in rare lymphocytes around capillaries on the papillary dermis of CC (**D1**, IHCx40) and large numbers of lymphocytes in the periphery granuloma TT (**D2**, HCI x40). CD27 expression in rare lymphocytes around capillaries in the papillary dermis of CC (**E1**, IHC x100) and large numbers of lymphocytes in the periphery and inside some TT granulomas (**E2**, IHCx40). No expression of CD52 in lymphocytes of CC for (**F1**, IHCx40) and large numbers of lymphocytes in the periphery of R1 granuloma (**F2**, IHC x40). EML2 expression absent in all skin components in DC (**G1**, X4 IHC) and weak positivity in macrophages of R2 (**G2**, IHCx40). FA2H expressed in sebaceous glands of CC (**H1**, IHCx10) and macrophage of R1 granulomas (**H2**, IHCx 40). GZMB expression absent in perivascular lymphocyte of CC (**I1**, IHCx40) and finely granular and cytoplasmic positivity in rare lymphocytes in the periphery and inside TT granulomas (**I2**, IHCx100). Strong LIPA expression in sebaceous glands of CC (**J1**, IHC x2) and macrophages of TT granulomas (**J2**, IHC x 40). Strong MMP9 expression in macrophages of TT and R2 granulomas (**K1**, IHCx40, **K2**, IHCx40). Absent expression of NCF1 in all skin components in CC (**L1**, IHCx2) and positivity in macrophage of R2 granuloma (**L2**, IHCx 40). Weak immunostaining of PTX3 on perivascular cells in CC (**M1**, IHCx20) and in macrophages, neutrophils, and interstitial cells of R2 (**M2**, IHCx 40). SIGLEC15 expression in endothelial cells of CC (**N1**, IHCx 20) and macrophages in LL (**N2**, IHCx 40). UBD expression in the inner layer of the excretory duct of sweat gland cells (**O1**, IHCx 4) and in vacuoles of macrophage in LL (**O2**, IHCx 100).

Evaluation of marker expression by IHC is detailed in Supplement 20. Figures 2 and 3 consist of two images showing the histology of the skin sample panel representing the histological patterns of the clinical forms and reactional states, and the expression of these markers in some forms of the leprosy spectrum, reaction forms, and control. In summary: (1) ANGPTL4: CC showed cytoplasmic immunostaining in neural branches and apical granules of the inner-layer of the sweat glands. Macrophages in TT and BT granulomas showed intense positive staining. Weak or moderate expression was observed in BB and R1 granulomas. Weak staining or complete absence of staining was observed in macrophages of BL, LL, and R2. (2) BAI1: it was expressed in neural branches and CC sweat ducts. Granulomas in all forms and reactions lacked the expression of BAI1. (3) BCAT1: weak expression was observed in neural branches, sebaceous glands, and sweat glands of CC. There was intense positivity in macrophages of TT, BT, and R1 and moderate to strong expression in macrophages of granulomas in BB, BL, LL, and R2. (4) CD2: expression was observed only in rare lymphocytes around vessels in the papillary dermis of CC. In the TT, BT, and R1 forms, a large number of lymphocytes in the periphery and within granulomas, perivascular space, nerves, and interstitium showed expression of CD2. In BB, BL, LL, and R2 few lymphocytes showed positivity. (5) CD27: there were rare CD27+ lymphocytes around capillaries in the superficial dermis of CC. In BT and TT, there were a large number of lymphocytes distributed mainly in the periphery of granulomas. In the BB, BL, and LL forms, there were fewer lymphocytes and homogeneous distribution was observed in the granulomas around the macrophages. In R1, lymphocytes were predominantly distributed in the periphery of the granulomas and were rare or absent in the center of reactional granulomas. In R2, there were no CD27+ lymphocytes within the abscess and there were a few within non-reactional granulomas. (6) CD52: immunostaining was observed in all skin components of CC, but rare lymphocytes around vessels in the papillary dermis were positively stained. CD52 expression was observed in lymphocytes surrounding granulomas, with larger numbers of lymphocytes in the TT, BT, and R1 granulomas, as well as lymphocytes infiltrating vessels, nerves, interstitial, arrector pili muscle, and epidermis. In BB, BL, LL, and R2 granulomas there were fewer CD52+ lymphocytes. (7) EML2: no expression was observed in all CC skin components. Positivity was observed in rare macrophages of granulomas in all forms and R1. Weak expression was observed in macrophages of R2 (8) FA2H: in CC intense positivity was seen in the sebaceous glands with absence in other components. There was moderate to intense positivity in macrophages constituting the TT granulomas and R1. In BT, BB, BL, LL, and R2 macrophages showed weak or moderate positivity. (9) GZMB: no expression was observed in CC. Fine granular cytoplasmic staining was observed in rare lymphocytes in the periphery or within the granulomas in all forms and reactional states, with a slight predominance in the TT group (10) LIPA: there was intense expression in the sebaceous glands and in the secretory cells of the sweat glands of CC. In TT, BT, and R1, moderate to severe positivity was observed in macrophages in the center of the granuloma. Expression was mild to moderate in macrophages of BB, BL, LL, and R2. (11) MMP9: In CC, rare perivascular macrophages were positive. In TT, BT, BB, BL, LL, and R1 intense expression was observed in macrophages. In R2, there was strong positivity in macrophages and neutrophils. Interstitial fibroblasts were positive in both reactional states. (12) NCF1: there was no expression in CC. In leprotic samples, weak to moderate positivity was observed in the cytoplasm of macrophages in all forms and reactions (13) PTX3: in CC, there was mild and focal expression on endothelial cells, neural branches, and some mononuclear cells around capillaries. Staining was present in all leprotic samples, especially on lymphocytes that composed the granulomas. In R2, there was expression in neutrophils and intersticial cells (14) SIGLEC15: the endothelium was positive in melanocytic cells in the basal layer of the epidermis in CC. In the leprotic samples, it was expressed in macrophages in all forms and reactional states. (16) UBD: expression was observed in cells of the inner portion of the sweat gland excretory duct cells in CC. In leprotic cases, weak and focal expression was observed in macrophages of granulomas of all forms and reactional states, especially in vacuoles inside the LL macrophages.

**Figure 4:**
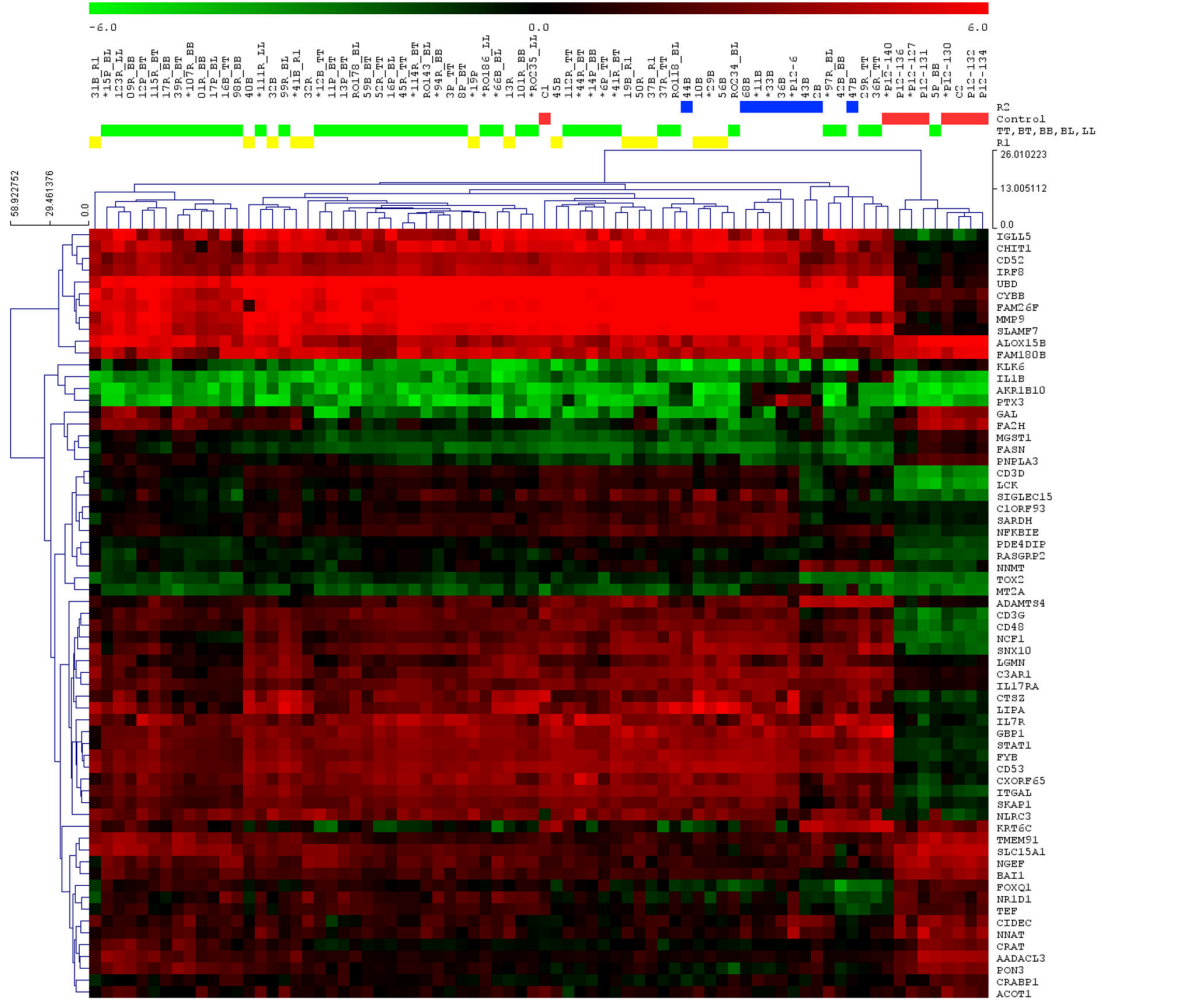
**Heat map of the 88 mRNAs submitted to RT-PCR validation**. Hierarchical unsupervised cluster, with euclidean distance and average linkage of all samples and differentially expressed gene validated. All samples [controls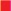, clinical forms (TT, BT, BB, BL, LL)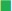, R1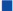, and R2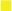] and the 69 genes validated were submitted to analysis of the unsupervised hierarchical groups, with correlation Pearson metric and average linkage. Samples selected for validation are marked with a ^*^.

## Discussion

Analysis of mRNA expression in leprotic skin lesions is complex since differentially expressed values may vary because of changes resulting from the disease process and differences in histological patterns of skin segments. With regard to leprotic lesions, there are important variations in the extension of inflammatory infiltration and cellular composition of granulomas usually associated with different histopathological characteristics of clinical forms, duration of the disease, and the intensity and development of reactional states (beginning, well established, or resolution phase) (Ridley and Jopling, 1966; Hastings et al., 1988). Furthermore, skin samples with leprosy may present with a decrease or disappearance of normal skin structures such as pilo sebaceous follicles, glands, neural branches, and arrector pili muscles. Thus, these important variables are difficult to standardize and can change the RNA expression profile, making it difficult to interpret the data. However, considering that samples were obtained from sites with similar histological patterns (trunk and limbs) is likely that most of the differentially expressed mRNAs are related to the pathophysiological mechanisms of leprosy.

The large number of differentially expressed mRNAs found between disease samples and healthy controls evidence the complexity of the pathophysiological mechanisms underlying leprosy at the molecular level and indicate that numerous genes are involved in establishment of the clinical forms, besides onset and progression of the reactional forms. It is clear that a large number of these mRNAs are present in all forms of the disease spectrum, in spite of variation in levels of expression, suggesting that most of them comprise a common pathophysiological mechanism of the disease. However, some mRNAs are exclusively expressed in certain forms or specific reactional types. Others, such as GJB2 and KRT6C, showed inverted expression, i.e., they were up-regulated in the clinical forms of the disease, but were down-regulated in the reactional states, or vice versa. These variations of expression are compatible with different clinic pathological characteristics of leprosy. Being an infectious disease, leprotic lesions are composed mainly of immune cells at various degrees of differentiation and therefore present with many mRNAs common to inflammatory and/or infectious processes. Furthermore, the cellular composition of granulomas has some peculiarities, which are characterized by distinct clinic pathological patterns between the polar groups [(TT + BT) vs. (LL + BL)] and the reactional forms (R1 vs. R2). Granulomas on the tuberculoid side are well defined and composed of epithelioid macrophages (M1 profile) surrounded mainly by large numbers of T and B lymphocytes and other cells. On the lepromatous side, granulomas are diffuse, with vacuolated macrophages (M2 profile) and scarce lymphocytic infiltrates. The reactions also have significant differences in cellular composition. In all type “1” reactional lesions, there is an influx of lymphocytes and young macrophages (M1 profile) resulting in disorganization of pre-existing granulomas, edema, and sometimes necrosis. In type “2” reaction, neutrophils, which are almost absent in the clinical forms and in the type “1” reaction, are a major component and commonly form microabscesses (Figure 2). In both reactional states, vessels and stromal cells undergo important changes. Therefore, these differences are probably related to particular expression of some mRNAs in the clinical forms and reactional episodes.

The complexity of leprosy at the molecular level is also observed in the numerous signaling pathways involved in the disease and in the reactional states. The large number of differentially expressed mRNAs detected in this study, are also differentially expressed in a number of diseases such as various cancers (breast, colon, prostate, liver, pancreas, kidney, central nervous system, etc.), storage diseases, inflammatory/autoimmune diseases (Crohn's disease, psoriasis, ulcerative colitis, and systemic lupus erythematosus), neurodegenerative (Alzheimer's disease), infectious diseases (tuberculosis, other mycobacterial infections and AIDS), non-infectious granulomatous disease, and others (diabetes and atherosclerosis). This indicates that leprosy shares pathophysiologic mechanisms with a large number of non-neoplastic and neoplastic diseases (Supplement 21). This explains in part why leprosy can clinically mimic a large number of these diseases with overlapping clinical and pathological features and similar expression of these laboratory markers, which makes it difficult to diagnose and delays diagnosis for long periods (Foss and Motta, 2012). For example, leprosy commonly mimics certain autoimmune diseases such as systemic lupus erythematosus, psoriasis, and arthritis (Prasad et al., 2013). Data from literature shows that the mRNAs that we subjected to RT-PCR validation are related to numerous pathophysiological processes, mainly immune response, cell proliferation, apoptosis, angiogenesis, matrix extracellular, damage, and/or neural repair and lipid metabolism. These processes are regulated by important signaling pathways. Several of these mRNAs are involved in regulating the immune response, for example, NF-κB, TGF-beta superfamily, IL-17, and p53 signaling are involved in antigen presentation (Supplements 16–18 and 21).

Molecular studies have identified numerous markers that participate in the pathological processes of cancer and infectious or inflammatory diseases. These molecules have been the subject of experimental studies and have also been tested in several clinical studies. New drugs, such as monoclonal antibodies (biological) are in testing phase or have already been applied for the treatment of various diseases (Supplement 21). Even though important metabolic disorders are caused by such drugs, studies carried out in cancer, degenerative diseases, and metabolic, infectious, and genetic disorders, can also contribute to the discovery of alternative new forms of treatment for leprosy and its reactional states, based on the assumption that several mRNAs differentially expressed in leprosy are therapeutic targets for these diseases. However, specific studies need to be conducted to evaluate the actual value of these mRNAs in the context of leprosy. Interestingly, some drugs such as clofazimine and thalidomide, which are commonly used in leprosy, are also used for the treatment of neoplastic and non-neoplastic diseases (Zhou et al., 2013; Koval et al., 2014). Studies demonstrated that clofazimine can inhibit the cell growth in triple-negative breast cancer (Koval et al., 2014). Thalidomide, a drug commonly used to treat type “2” (R2) reactional episodes has been successfully employed in the treatment of a large number of diseases, including inflammatory diseases and cancers, such as myeloma (Zhou et al., 2013).

There are few reports in literature on mRNA expression in leprosy. In general, studies have shown that genes associated with negative regulation of the immune system are also associated with leprosy (Liu et al., 2012; Guerreiro et al., 2013; Orlova et al., 2013; Singh et al., 2013). Some of these previously described mRNAs, such as ubiquitin, are also differentially expressed in the present study (Mehta and Liu, 2014). Ubiquitination is an important biochemical process that controls many aspects of protein function such as degradation and protein-protein interaction. It is also involved in induction of apoptosis, cell cycle control, and activation of NF-κB (nuclear factor kappa enhancer binding protein). The transcription factor NF-κB, in turn, controls many processes including immunity, inflammation, and apoptosis. The UBD gene was found to be highly expressed in leprosy, indicating that the ubiquitination is a common process across the spectrum of the disease, but with higher values in type 1 reaction (Figure 3O1 and Supplement 19).

Bleharski et al. (2003) evaluated the genes expression in skin lesions of leprosy patients with the polar forms (six T-lep and five L-lep), diagnosed according to R&J criteria. They identified several mRNAs up regulated in T-lep associated with antigen processing/presentation (UBD, PSMB4, CD1b, CD28, and CD79a), anti-microbial (cathepsin g and SLP1) and pro-inflammatory/Th1 (SLAM, Il-1 receptor, IL-15, IL-7, IL12 receptor-beta e lymphotoxin-α). In the L-lep the up regulated genes were associated with anti-inflammatory/Th2 (TGFß1, IL-5, and latent TGFß protein-2), inhibitory receptors (SIRP-1α, LIR-7, LIR-4, LIR3 e LIR8) and B cell response (CD80, Ig heavy chain gamma 3, CD83, Ig kappa chain, anti-colorretal carcinoma heavy chain, MD-1, CD22, BLNK, rearranged Ig heavy chain, Ig lambda locus, Ig kappa light chain variable region, CD19, Ig heavy chain constant M, TNFRSF17, IgM binding protein 2 and Ig lambda-like polypeptide 1). In the present study, the comparison between TT and LL (Supplement 4) did not evidence the same differentially expressed mRNAs, however, some of these mRNAs were identified in other comparisons (UBD, SLP1, SLAM, IL15, TGFß1, CD83, and CD19) like disease vs. CC (Supplement 2). Other mRNAs identified by Bleharski et al. (2003), though not related to the mentioned pathways, were identified in the present study (CD47, FcER1 gamma, IFNα receptor 1, CD14, chitinase 1, and CD4).

Siddiqui et al., identified in chromosome 10p13 a locus associated to susceptibility to leprosy in Indian patients (Siddiqui et al., 2001). In the present study, 2 genes differentially expressed that are present in the chromosome 10p13 (CCDC3 down-regulated and CELF2 up-regulated), were observed comparing disease vs. CC. In the literature, the Coiled-coil domain containing 3 (CCDC3) represses tumor necrosis factor-α/nuclear factor κB-induced endothelial inflammation and the CELF2 is associated with T-cell signaling and it is also a suppressor gene of colon cancer (Ramalingam et al., 2012; Azad et al., 2014; Mallory et al., 2015). Studies about the function of both genes in leprosy are unknown.

Evaluation of protein expression of 15 selected genes by IHC showed that there was agreement in most of the samples with the microarray analysis. The same results were obtained for most up or down-regulated mRNAs detected by the microarray and validated by RT-PCR and in the detection or lack of protein expression in the inflammatory infiltrates of leprosy clinical forms and reaction states (Figure 3). However, some genes (UBD and GZMB, for example) showed disagreement, where up-regulation of mRNA was observed in the microarray and RT-PCR, weak immunostaining or focal inflammatory processes were seen in leprotic samples subject to IHC. Therefore, small changes or even conflicting data between microarray gene expression/RT-PCR and IHC indicates that other regulatory mechanisms are probably involved in the pathophysiological processes of leprosy.

In summary, this study shows that leprosy is a complex disease from the molecular point of view, since a large number of mRNAs involved in several pathophysiological mechanisms and signaling pathways are differentially expressed in leprosy related mainly to antigen presentation pathway, cytotoxic T lymphocyte-mediated apoptosis of target cells, and B cell development. These numerous mRNAs, which are probably involved in the establishment and progression of the clinical forms of leprosy and in the reactional episodes, are also reported in numerous other diseases. These findings open new perspectives that may be used in the development of new approaches to treat different diseases and identification of biomarkers with predictive or therapeutic roles in leprosy.

## Author contributions

CS, AB, DC, FS Conceived the project. CG, SU, JB, CS Clinical evaluation of patients and Biopsy procedures. CS Histopathological and immunohistochemical analysis. AB, AC, MP, AT, LF RNA samples preparation, hybridization, and PCR. MP, AC, AB, AT, LF, PR, CS Data analysis. CS, AB, PR Wrote the manuscript. All authors critically revised the paper for intellectual content.

## Funding

This work was funded by the São Paulo State Research Foundation (FAPESP) Process no. 2010/19286-3.

### Conflict of interest statement

The authors declare that the research was conducted in the absence of any commercial or financial relationships that could be construed as a potential conflict of interest.

## References

[B1] Akama T. Suzuki K. Tanigawa K. Kawashima A. Wu H. Nakata N.. (2009). Whole-genome tiling array analysis of *Mycobacterium leprae* RNA reveals high expression of pseudogenes and noncoding regions. J. Bacteriol. 191, 3321–3327. 10.1128/JB.00120-09 19286800 PMC2687151

[B2] Azad A. K. Chakrabarti S. Xu Z. Davidge S. T. Fu Y. (2014). Coiled-coildomaincontaining3 (CCDC3) represses tumor necrosis factor- α/nuclear factor κB-induced endothelial inflammation. Cell. Signal. 26, 2793–2800. 10.1016/j.cellsig.2014.08.025 25193116

[B3] Bleharski J. R. Li H. Meinken C. Graeber T. G. Ochoa M. T. Yamamura M.. (2003). Use of genetic profiling in leprosy to discriminate clinical forms of the disease. Science 301, 1527–1530. 10.1126/science.1087785 12970564

[B4] Foss N. T. Motta A. C. (2012). Leprosy, a neglected disease that causes a wide variety of clinical conditions in tropical countries. Mem. Inst. Oswaldo Cruz 107, 28–33. 10.1590/S0074-02762012000900006 23283450

[B5] França R. F. da Silva C. C. De Paula S. O. (2013). Recent advances in molecular medicine techniques for the diagnosis, prevention, and control of infectious diseases. Eur. J. Clin. Microbiol. Infect. Dis. 32, 723–728. 10.1007/s10096-013-1813-0 23339016 PMC7087945

[B6] Guerreiro L. T. Robottom-Ferreira A. B. Ribeiro-Alves M. Toledo-Pinto T. G. Rosa Brito T. Rosa P. S.. (2013). Gene expression profiling specifies chemokine, mitochondrial and lipid metabolism signatures in leprosy. PLoS ONE 8:e64748. 10.1371/journal.pone.0064748 23798993 PMC3683049

[B7] Hastings R. C. Gillis T. P. Krahenbuhl J. L. Franzblau S. G. (1988). Leprosy. Clin. Microbiol. Rev. 1, 330–348. 3058299 10.1128/cmr.1.3.330 PMC358054

[B8] Koval A. V. Vlasov P. Shichkova P. Khunderyakova S. Markov Y. Panchenko J.. (2014). Anti-leprosy drug clofazimine inhibits growth of triple-negative breast cancer cells via inhibition of canonical Wnt signaling. Biochem. Pharmacol. 87, 571–578. 10.1016/j.bcp.2013.12.007 24355563

[B9] Lam S. W. Jimenez C. R. Boven E. (2014). Breast cancer classification by proteomic technologies: current state of knowledge. Cancer Treat Ver 40, 129–138. 10.1016/j.ctrv.2013.06.006 23891266

[B10] Liu P. T. Wheelwright M. Teles R. Komisopoulou E. Edfeldt K. Ferguson B.. (2012). MicroRNA-21 targets the vitamin D-dependent antimicrobial pathway in leprosy. Nat. Med. 18, 267–273. 10.1038/nm.2584 22286305 PMC3274599

[B11] Mallory M. J. Allon S. J. Qiu J. Gazzara M. R. Tapescu I. Martinez N. M.. (2015). Induced transcription and stability of CELF2 mRNA drives widespread alternative splicing during T-cell signaling. Proc. Natl. Acad. Sci. U.S.A. 112, E2139–E2148. 10.1073/pnas.1423695112 25870297 PMC4418860

[B12] Mehta M. D. Liu P. T. (2014). microRNAs in mycobacterial disease: friend or foe? Front. Genet. 5:231. 10.3389/fgene.2014.00231 25076967 PMC4097432

[B13] Novoradovskaya N. Whitfield M. L. Basehore L. S. Novoradovsky A. Pesich R. Usary J.. (2004). Universal reference RNA as a standard for microarray experiments. BMC Genomics 5:20. 10.1186/1471-2164-5-20 15113400 PMC394318

[B14] Orlova M. Cobat A. Huong N. T. Ba N. N. Van Thuc N. Spencer J.. (2013). Gene set signature of reversal reaction type I in leprosy patients. PLoS Genet. 9:e1003624. 10.1371/journal.pgen.1003624 23874223 PMC3708838

[B15] Pasic M. D. Samaan S. Yousef G. M. (2013). Genomic medicine: new frontiers and new challenges. Clin. Chem. 59, 158–167. 10.1373/clinchem.2012.184622 23284016

[B16] Prasad S. Misra R. Aggarwal A. Lawrence A. Haroon N. Wakhlu A.. (2013). Leprosy revealed in a rheumatology clinic: a case series. Int. J. Rheum. Dis. 16, 129–133. 10.1111/j.1756-185X.2012.01819.x 23773635

[B17] Ramalingam S. Ramamoorthy P. Subramaniam D. Anant S. (2012). Reduced expression of RNA binding protein CELF2, a putative tumor suppressor gene in colon cancer. Immunogastroenterology 1, 27–33. 10.7178/ig.1.1.7 23795348 PMC3686119

[B18] Ridley D. S. Jopling W. H. (1966). Classification of leprosy according to immunity. A five-group system. Int. J. Lepr. Other Mycobact. Dis. 34, 255–273. 5950347

[B19] Sethi S. Ali S. Philip P. A. Sarkar F. H. (2013). Clinical advances in molecular biomarkers for cancer diagnosis and therapy. Int. J. Mol. Sci. 14, 14771–14784. 10.3390/ijms140714771 23863689 PMC3742272

[B20] Siddiqui M. R. Meisner S. Tosh K. Balakrishnan K. Ghei S. Fisher S. E.. (2001). A major susceptibility locus for leprosy in India maps to chromosome 10p13. Nat. Genet. 27, 439–441. 10.1038/86958 11279529

[B21] Singh P. K. Singh A. V. Chauhan D. S. (2013). Current understanding on micro RNAs and its regulation in response to Mycobacterial infections. J. Biomed. Sci. 20:14. 10.1186/1423-0127-20-14 23448104 PMC3599176

[B22] Vandesompele J. De Preter K. Pattyn F. Poppe B. Van Roy N. De Paepe A.. (2002). Accurate normalization of real-time quantitative RT-PCR data by geometric averaging of multiple internal control genes. Genome Biol. 3:RESEARCH0034. 12184808 10.1186/gb-2002-3-7-research0034 PMC126239

[B23] World Health Organization (2014). Global leprosy update, 2013; reducing disease burden. Wkly. Epidemiol. Rec. 89, 389–400. 25202781

[B24] Zhou S. Wang F. Hsieh T. C. Wu J. M. Wu E. (2013). Thalidomide-a notorious sedative to a wonder anticancer drug. Curr. Med. Chem. 20, 4102–4108. 10.2174/09298673113209990198 23931282 PMC4112512

