# Supplementary material for: Genome-Wide Screening of mRNA Expression in Leprosy Patients
Source: Front Genet. 2015 Nov 20;6:334. doi: 10.3389/fgene.2015.00334 (PMC4653304; doi:10.3389/fgene.2015.00334)
Supplement: Supplementary file 6 [file Table6.DOCX]

| **Marker** | **CC** | **TT** | **BT** | **BB** | **BL** | **LL** | **R1** | **R2** |
| --- | --- | --- | --- | --- | --- | --- | --- | --- |
| **ANGPTL4** | Granuloma: (none)  Nerve: (+)  Schwann cells  Vessels : (-)  Fibroblast: (-)  Pili muscle: (-)  Epidermis: (-)  Adnexal: (+)  Sweet glands | Granuloma: (+++)  Macrophage(+++)  Nerve: (+)  Schwann cells(+)  Vessels : (-)  Fibroblast: (-)  Pili muscle: (-)  Epidermis: (-)  Adnexal: (+)  Sweet glands | Granuloma: (+++)  Macrophage(+++)  Nerve: (+)  Schwann cells(+)  Vessels : (-)  Fibroblast: (-)  Pili muscle: (-)  Epidermis: (-)  Adnexal: (+)  Sweet glands | Granuloma: (+)  Macrophage(+)  Nerve: (+)  Schwann cells(+)  Vessels : (-)  Fibroblast: (-)  Pili muscle: (-)  Epidermis: (-)  Adnexal: (+)  Sweet glands | Granuloma: (+)  Macrophage(+)  Nerve: (+)  Schwann cells(+)  Vessels : (-)  Fibroblast: (-)  Pili muscle: (-)  Epidermis: (-)  Adnexal: (+)  Sweet glands | Granuloma: (+)  Macrophage(+)  Nerve: (+)  Schwann cells(+)  Vessels : (-)  Fibroblast: (-)  Pili muscle: (-)  Epidermis: (-)  Adnexal: (+)  Sweet glands | Granuloma: (++)  Macrophage(++)  Nerve: (+)  Schwann cells(+)  Vessels : (-)  Fibroblast: (-)  Pili muscle: (-)  Epidermis: (-)  Adnexal: (+)  Sweet glands | Granuloma: (+)  Macrophage(+)  Nerve: (+)  Schwann cells(+)  Vessels : (-)  Fibroblast: (-)  Pili muscle: (-)  Epidermis: (-)  Adnexal: (+)  Sweet glands |
| **BAI1** | Granuloma: (none)  Nerve: (+)  Schwann cells(+)  Vessels : (-)  Fibroblast: (-)  Pili muscle: (-)  Epidermis: (-)  Adnexal: (+)  folículo(+) | Granuloma: (-)  Nerve: (+)  Schwann cells(+)  Vessels : (-)  Fibroblast: (-)  Pili muscle: (-)  Epidermis: (-)  Adnexal: (+)  folículo(+) | Granuloma: (-)  Nerve: (+)  Schwann cells(+)  Vessels : (-)  Fibroblast: (-)  Pili muscle: (-)  Epidermis: (-)  Adnexal: (+)  folículo(+) | Granuloma: (-)    Nerve: (+)  Schwann cells(+)  Vessels : (-)  Fibroblast: (-)  Pili muscle: (-)  Epidermis: (-)  Adnexal: (+)  folículo(+) | Granuloma: (-)  Nerve: (+)  Schwann cells(+)  Vessels : (-)  Fibroblast: (-)  Pili muscle: (-)  Epidermis: (-)  Adnexal: (+)  folículo(+) | Granuloma: (-)    Nerve: (+)  Schwann cells(+)  Vessels : (-)  Fibroblast: (-)  Pili muscle: (-)  Epidermis: (-)  Adnexal: (+)  folículo(+) | Granuloma: (-)  Nerve: (+)  Schwann cells(+)  Vessels : (-)  Fibroblast: (-)  Pili muscle: (-)  Epidermis: (-)  Adnexal: (+)  folículo(+) | Granuloma: (-)    Nerve: (+)  Schwann cells(+)  Vessels : (-)  Fibroblast: (-)  Pili muscle: (-)  Epidermis: (-)  Adnexal: (+)  folículo(+) |
| **BCAT1** | Granuloma: (none)  Nerve: (+)  Vessels : (-)  Fibroblast: (-)  Pili muscle: (-)  Epidermis: (-)  Adnexal: (+)  folículo(+)  sweet gland(+) | Granuloma: (+++)  Macrophage(+++)  Nerve: (+)  Vessels : (-)  Fibroblast: (-)  Pili muscle: (-)  Epidermis: (-)  Adnexal: (+)  folículo(+)  sweet gland(+) | Granuloma: (+++)  Macrophage(+++)  Nerve: (+)  Vessels : (-)  Fibroblast: (-)  Pili muscle: (-)  Epidermis: (-)  Adnexal: (+)  folículo(+)  sweet gland(+) | Granuloma: (++)  Macrophage(++)  Nerve: (+)  Vessels : (-)  Fibroblast: (-)  Pili muscle: (-)  Epidermis: (-)  Adnexal: (+)  folículo(+)  sweet gland(+) | Granuloma: (++)  Macrophage(++)  Nerve: (+)  Vessels : (-)  Fibroblast: (-)  Pili muscle: (-)  Epidermis: (-)  Adnexal: (+)  folículo(+)  sweet gland(+) | Granuloma: (++)  Macrophage(++)  Nerve: (+)  Vessels : (-)  Fibroblast: (-)  Pili muscle: (-)  Epidermis: (-)  Adnexal: (+)  folículo(+)  sweet gland(+) | Granuloma: (+++)  Macrophage(+++)  Nerve: (+)  Vessels : (-)  Fibroblast: (-)  Pili muscle: (-)  Epidermis: (-)  Adnexal: (+)  folículo(+)  sweet gland(+) | Granuloma: (++)  Macrophage(++)  Neutrófilo(++)  Nerve: (+)  Vessels : (-)  Fibroblast: (-)  Pili muscle: (-)  Epidermis: (-)  Adnexal: (+)  folículo(+)  sweet gland(+) |
| **CD2** | Granuloma: (none)  Nerve: (-)  Vessels : (+)  Perivascular(+)  Fibroblast: (-)  Pili muscle: (-)  Epidermis: (-)  Adnexal: (-) | Granuloma: (+++)  linfócitos(+++)  Nerve: (+)  Vessels : (+)  Perivascular(+)  Fibroblast: (-)  Pili muscle: (+)  Epidermis: (+)  Adnexal: (-) | Granuloma: (+++)  linfócitos(+++)  Nerve: (+)  Vessels : (+)  Perivascular(+)  Fibroblast: (-)  Pili muscle: (+)  Epidermis: (+)  Adnexal: (-) | Granuloma: (++)  linfócitos(++)  Nerve: (+)  Vessels : (+)  Perivascular(+)  Fibroblast: (-)  Pili muscle: (+)  Epidermis: (+)  Adnexal: (-) | Granuloma: (++)  linfócitos(++)  Nerve: (+)  Vessels : (+)  Perivascular(+)  Fibroblast: (-)  Pili muscle: (+)  Epidermis: (+)  Adnexal: (-) | Granuloma: (++)  linfócitos(++)  Nerve: (+)  Vessels : (+)  Perivascular(+)  Fibroblast: (-)  Pili muscle: (+)  Epidermis: (+)  Adnexal: (-) | Granuloma: (++)  linfócitos(++)  Nerve: (+)  Vessels : (+)  Perivascular(+)  Fibroblast: (-)  Pili muscle: (+)  Epidermis: (+)  Adnexal: (-) | Granuloma: (++)  linfócitos(++)  Nerve: (+)  Vessels : (+)  Perivascular(+)  Fibroblast: (-)  Pili muscle: (+)  Epidermis: (+)  Adnexal: (-) |
| **CD27** | Granuloma: (none)  Nerve: (-)  Vessels : (+)  Perivascular(+)  Fibroblast: (-)  Pili muscle: (-)  Epidermis: (-)  Adnexal: (-) | Granuloma: (++)  linfócitos(++)  Nerve: (+)  Vessels : (+)  Fibroblast: (-)  Pili muscle: (+)  Epidermis: (+)  Adnexal: (-) | Granuloma: (++)  linfócitos(++)  Nerve: (+)  Vessels : (+)  Fibroblast: (-)  Pili muscle: (+)  Epidermis: (+)  Adnexal: (-) | Granuloma: (+)  linfócitos(+)  Nerve: (+)  Vessels : (+)  Fibroblast: (-)  Pili muscle: (+)  Epidermis: (+)  Adnexal: (-) | Granuloma: (+)  linfócitos(+)  Nerve: (+)  Vessels : (+)  Fibroblast: (-)  Pili muscle: (+)  Epidermis: (+)  Adnexal: (-) | Granuloma: (+)  linfócitos(+)  Nerve: (+)  Vessels : (+)  Fibroblast: (-)  Pili muscle: (+)  Epidermis: (+)  Adnexal: (-) | Granuloma: (+)  linfócitos(+)  Nerve: (+)  Vessels : (+)  Fibroblast: (-)  Pili muscle: (+)  Epidermis: (+)  Adnexal: (-) | Granuloma: (+)  linfócitos(+)  Nerve: (+)  Vessels : (+)  Fibroblast: (-)  Pili muscle: (+)  Epidermis: (+)  Adnexal: (-) |
| **CD52** | Granuloma: (none)  Nerve: (-)  Vessels : (-)*  Fibroblast: (-)  Pili muscle: (-)  Epidermis: (-)  Adnexal: (-) | Granuloma: (++) Lymphocite(++)  Nerve: (+)  Vessels : (+)  Fibroblast: (-)  Pili muscle: (+)  Epidermis: (+)  Adnexal: (-) | Granuloma: (++) Lymphocite(++)  Nerve: (+)  Vessels : (+)  Fibroblast: (-)  Pili muscle: (+)  Epidermis: (+)  Adnexal: (-) | Granuloma: (++) Lymphocite(++)  Nerve: (+)  Vessels : (+)  Fibroblast: (-)  Pili muscle: (+)  Epidermis: (+)  Adnexal: (-) | Granuloma: (++) Lymphocite(++)  Nerve: (+)  Vessels : (+)  Fibroblast: (-)  Pili muscle: (+)  Epidermis: (+)  Adnexal: (-) | Granuloma: (++) Lymphocite(++)  Nerve: (+)  Vessels : (+)  Fibroblast: (-)  Pili muscle: (+)  Epidermis: (+)  Adnexal: (-) | Granuloma: (++) Lymphocite(++)  Nerve: (+)  Vessels : (+)  Fibroblast: (-)  Pili muscle: (+)  Epidermis: (+)  Adnexal: (-) | Granuloma: (++) Lymphocite(++)  Nerve: (+)  Vessels : (+)  Fibroblast: (-)  Pili muscle: (+)  Epidermis: (+)  Adnexal: (-) |
| **EML2** | Granuloma: (none)  Nerve: (-)  Vessels : (-)  Fibroblast: (-)  Pili muscle: (-)  Epidermis: (-)  Adnexal: (-) | Granuloma: (0/+)  Macrophage(0/+)  Nerve: (-)  Vessels : (-)  Fibroblast: (-)  Pili muscle: (-)  Epidermis: (-)  Adnexal: (-) | Granuloma: (0/+)  Macrophage(0/+)  Nerve: (-)  Vessels : (-)  Fibroblast: (-)  Pili muscle: (-)  Epidermis: (-)  Adnexal: (-) | Granuloma: (0/+)  Macrophage(0/+)  Nerve: (-)  Vessels : (-)  Fibroblast: (-)  Pili muscle: (-)  Epidermis: (-)  Adnexal: (-) | Granuloma: (0/+)  Macrophage(0/+)  Nerve: (-)  Vessels : (-)  Fibroblast: (-)  Pili muscle: (-)  Epidermis: (-)  Adnexal: (-) | Granuloma: (0/+)  Macrophage(0/+)  Nerve: (-)  Vessels : (-)  Fibroblast: (-)  Pili muscle: (-)  Epidermis: (-)  Adnexal: (-) | Granuloma: (0/+)  Macrophage(0/+)  Nerve: (-)  Vessels : (-)  Fibroblast: (-)  Pili muscle: (-)  Epidermis: (-)  Adnexal: (-) | Granuloma: (+/++)  Macrophage(+/++)  Nerve: (-)  Vessels : (-)  Fibroblast: (-)  Pili muscle: (-)  Epidermis: (-)  Adnexal: (-) |
| **FA2H** | Granuloma: (none)  Nerve: (-)  Vessels : (-)  Fibroblast: (-)  Pili muscle: (-)  Epidermis: (-)  Adnexal: (+++)  Gland sebácea(+++) | Granuloma: (+++)  Macrophage(+++)  Nerve: (-)  Vessels : (-)  Fibroblast: (-)  Pili muscle: (-)  Epidermis: (-)  Adnexal: (+++)  Gland sebácea(+++) | Granuloma: (++)  Macrophage(++)  Nerve: (-)  Vessels : (-)  Fibroblast: (-)  Pili muscle: (-)  Epidermis: (-)  Adnexal: (+++)  Gland sebácea(+++) | Granuloma: (++)  Macrophage(++)  Nerve: (-)  Vessels : (-)  Fibroblast: (-)  Pili muscle: (-)  Epidermis: (-)  Adnexal: (+++)  Gland sebácea(+++) | Granuloma: (++)  Macrophage(++)  Nerve: (-)  Vessels : (-)  Fibroblast: (-)  Pili muscle: (-)  Epidermis: (-)  Adnexal: (+++)  Gland sebácea(+++) | Granuloma: (+++)  Macrophage(+++)  Nerve: (-)  Vessels : (-)  Fibroblast: (-)  Pili muscle: (-)  Epidermis: (-)  Adnexal: (+++)  Gland sebácea(+++) | Granuloma: (++)  Macrophage(++)  Nerve: (-)  Vessels : (-)  Fibroblast: (-)  Pili muscle: (-)  Epidermis: (-)  Adnexal: (+++)  Gland sebácea(+++) | Granuloma: (++)  Macrophage(++)  Nerve: (-)  Vessels : (-)  Fibroblast: (-)  Pili muscle: (-)  Epidermis: (-)  Adnexal: (+++)  Gland sebácea(+++) |
| **GZMB** | Granuloma: (none)  Nerve: (-)  Vessels : (-)  Fibroblast: (-)  Pili muscle: (-)  Epidermis: (-)  Adnexal: (-) | Granuloma: (+)  Nerve: (-)  Vessels : (-)  Fibroblast: (-)  Pili muscle: (-)  Epidermis: (-)  Adnexal: (-) | Granuloma: (+)  Nerve: (-)  Vessels : (-)  Fibroblast: (-)  Pili muscle: (-)  Epidermis: (-)  Adnexal: (-) | Granuloma: (+)  Nerve: (-)  Vessels : (-)  Fibroblast: (-)  Pili muscle: (-)  Epidermis: (-)  Adnexal: (-) | Granuloma: (+)  Nerve: (-)  Vessels : (-)  Fibroblast: (-)  Pili muscle: (-)  Epidermis: (-)  Adnexal: (-) | Granuloma: (+)  Nerve: (-)  Vessels : (-)  Fibroblast: (-)  Pili muscle: (-)  Epidermis: (-)  Adnexal: (-) | Granuloma: (+)  Nerve: (-)  Vessels : (-)  Fibroblast: (-)  Pili muscle: (-)  Epidermis: (-)  Adnexal: (-) | Granuloma: (+)  Nerve: (-)  Vessels : (-)  Fibroblast: (-)  Pili muscle: (-)  Epidermis: (-)  Adnexal: (-) |
| **LIPA** | Granuloma: (none)  Nerve: (-)  Vessels : (-)  Fibroblast: (-)  Pili muscle: (-)  Epidermis: (-)  Adnexal: (+++)  Gland sebácea  Gland sudorípara | Granuloma: (+++) Macrophage(+++)  Nerve: (-)  Vessels : (-)  Fibroblast: (-)  Pili muscle: (-)  Epidermis: (-)  Adnexal: (-) | Granuloma: (+++) Macrophage(+++)  Nerve: (-)  Vessels : (-)  Fibroblast: (-)  Pili muscle: (-)  Epidermis: (-)  Adnexal: (-) | Granuloma: (++) Macrophage(++)  Nerve: (-)  Vessels : (-)  Fibroblast: (-)  Pili muscle: (-)  Epidermis: (-)  Adnexal: (-) | Granuloma: (++) Macrophage(++)  Nerve: (-)  Vessels : (-)  Fibroblast: (-)  Pili muscle: (-)  Epidermis: (-)  Adnexal: (-) | Granuloma: (++) Macrophage(++)  Nerve: (-)  Vessels : (-)  Fibroblast: (-)  Pili muscle: (-)  Epidermis: (-)  Adnexal: (-) | Granuloma: (+++) Macrophage(+++)  Nerve: (-)  Vessels : (-)  Fibroblast: (-)  Pili muscle: (-)  Epidermis: (-)  Adnexal: (-) | Granuloma: (++) Macrophage(++)  Nerve: (-)  Vessels : (-)  Fibroblast: (-)  Pili muscle: (-)  Epidermis: (-)  Adnexal: (-) |
| **MMP9** | Granuloma: (none)  Nerve: (-)  Vessels : (-)  Macrófagos perivasculares (+)  Fibroblast: (-)  Pili muscle: (-)  Epidermis: (-)  Adnexal: (-) | Granuloma: (+++) Macrophage(+++)  Nerve: (-)  Vessels : (-)  Fibroblast: (-)  Pili muscle: (-)  Epidermis: (-)  Adnexal: (-) | Granuloma: (+++) Macrophage(+++)  Nerve: (-)  Vessels : (-)  Fibroblast: (-)  Pili muscle: (-)  Epidermis: (-)  Adnexal: (-) | Granuloma: (+++) Macrophage(+++)  Nerve: (-)  Vessels : (-)  Fibroblast: (-)  Pili muscle: (-)  Epidermis: (-)  Adnexal: (-) | Granuloma: (+++) Macrophage(+++)  Nerve: (-)  Vessels : (-)  Fibroblast: (-)  Pili muscle: (-)  Epidermis: (-)  Adnexal: (-) | Granuloma: (+++) Macrophage(+++)  Nerve: (-)  Vessels : (-)  Fibroblast: (-)  Pili muscle: (-)  Epidermis: (-)  Adnexal: (-) | Granuloma: (+++) Macrophage(+++)  Nerve: (-)  Vessels : (-)  Fibroblast: (-)  Pili muscle: (-)  Epidermis: (-)  Adnexal: (-) | Granuloma: (+++) Macrophage(+++)  Neutrófilos (+++)  Nerve: (-)  Vessels : (-)  Fibroblast: (-)  Pili muscle: (-)  Epidermis: (-)  Adnexal: (-) |
| **NCF1** | Granuloma: (none)  Nerve: (-)  Vessels : (-)  Fibroblast: (-)  Pili muscle: (-)  Epidermis: (-)  Adnexal: (-) | Granuloma: (+)  Macrophage(+)  Nerve: (-)  Vessels : (-)  Fibroblast: (-)  Pili muscle: (-)  Epidermis: (-)  Adnexal: (-) | Granuloma: (+)  Macrophage(+)  Nerve: (-)  Vessels : (-)  Fibroblast: (-)  Pili muscle: (-)  Epidermis: (-)  Adnexal: (-) | Granuloma: (+)  Macrophage(+)  Nerve: (-)  Vessels : (-)  Fibroblast: (-)  Pili muscle: (-)  Epidermis: (-)  Adnexal: (-) | Granuloma: (+)  Macrophage(+)  Nerve: (-)  Vessels : (-)  Fibroblast: (-)  Pili muscle: (-)  Epidermis: (-)  Adnexal: (-) | Granuloma: (+)  Macrophage(+)  Nerve: (-)  Vessels : (-)  Fibroblast: (-)  Pili muscle: (-)  Epidermis: (-)  Adnexal: (-) | Granuloma: (+)  Macrophage(+)  Nerve: (-)  Vessels : (-)  Fibroblast: (-)  Pili muscle: (-)  Epidermis: (-)  Adnexal: (-) | Granuloma: (+)  Macrophage(+)  Nerve: (-)  Vessels : (-)  Fibroblast: (-)  Pili muscle: (-)  Epidermis: (-)  Adnexal: (-) |
| **PTX3** | Granuloma: (none)  Nerve: (+)  Vessels : (+)  Endotélio (+)  Fibroblast: (-)  Pili muscle: (-)  Epidermis: (-)  Adnexal: (-) | Granuloma: (+/++)  linfocitos(+/++)  Nerve: (+)  Vessels : (+)  Endotélio (+)  Fibroblast: (-)  Pili muscle: (-)  Epidermis: (-)  Adnexal: (-) | Granuloma: (+/++)  linfocitos(+/++)  Nerve: (+)  Vessels : (+)  Endotélio (+)  Fibroblast: (-)  Pili muscle: (-)  Epidermis: (-)  Adnexal: (-) | Granuloma: (+/++)  linfocitos(+/++)  Nerve: (+)  Vessels : (+)  Endotélio (+)  Fibroblast: (-)  Pili muscle: (-)  Epidermis: (-)  Adnexal: (-) | Granuloma: (+/++)  linfocitos(+/++)  Nerve: (+)  Vessels : (+)  Endotélio (+)  Fibroblast: (-)  Pili muscle: (-)  Epidermis: (-)  Adnexal: (-) | Granuloma: (+/++)  linfocitos(+/++)  Nerve: (+)  Vessels : (+)  Endotélio (+)  Fibroblast: (-)  Pili muscle: (-)  Epidermis: (-)  Adnexal: (-) | Granuloma: ()  Granuloma: (+/++)  linfocitos(+/++)  Nerve: (+)  Vessels : (+)  Endotélio (+)  Fibroblast: (-)  Pili muscle: (-)  Epidermis: (-)  Adnexal: (-) | Granuloma: (++/+++)  linfócitos(++/+++)  Nerve: (+)  Vessels : (+)  Fibroblast: (+++)  Interstício (+++)  Pili muscle: (-)  Epidermis: (-)  Adnexal: (-) |
| **SIGLEC15** | Granuloma: (none)  Nerve: (-)  Vessels : (+)  Endotélio(+)  Fibroblast: (-)  Pili muscle: (-)  Epidermis: (+)  melanocito(+)  Adnexal: ) | Granuloma: (+/++)  Macrophage(+/++)  Nerve: (-)  Vessels : (+)  Endotélio(+)  Fibroblast: (-)  Pili muscle: (-)  Epidermis: (+)  Melanocito(+)  Adnexal: (-) | Granuloma: ()  Granuloma: (+/++)  Macrophage(+/++)  Nerve: (-)  Vessels : (+)  Endotélio(+)  Fibroblast: (-)  Pili muscle: (-)  Epidermis: (+)  Melanocito(+)  Adnexal: (-) | Granuloma: (+/++)  Macrophage(+/++)  Nerve: (-)  Vessels : (+)  Endotélio(+)  Fibroblast: (-)  Pili muscle: (-)  Epidermis: (+)  Melanocito(+)  Adnexal: (-) | Granuloma: (+/++)  Macrophage(+/++)  Nerve: (-)  Vessels : (+)  Endotélio(+)  Fibroblast: (-)  Pili muscle: (-)  Epidermis: (+)  Melanocito(+)  Adnexal: (-) | Granuloma: (+/++)  Macrophage(+/++)  Nerve: (-)  Vessels : (+)  Endotélio(+)  Fibroblast: (-)  Pili muscle: (-)  Epidermis: (+)  Melanocito(+)  Adnexal: (-) | Granuloma: (+/++)  Macrophage(+/++)  Nerve: (-)  Vessels : (+)  Endotélio(+)  Fibroblast: (-)  Pili muscle: (-)  Epidermis: (+)  Melanocito(+)  Adnexal: (-) | Granuloma: (+/++)  Macrophage(+/++)  Nerve: (-)  Vessels : (+)  Endotélio(+)  Fibroblast: (-)  Pili muscle: (-)  Epidermis: (+)  Melanocito(+)  Adnexal: (-) |
| **UBD** | Granuloma: (none)  Nerve: (-)  Vessels : (-)  Fibroblast: (-)  Pili muscle: (-)  Epidermis: (-)  Adnexal: (+)  ducto sudoríparo | Granuloma: (0/+)  Macrophage(0/1)  Nerve: (-)  Vessels : (-)  Fibroblast: (-)  Pili muscle: (-)  Epidermis: (-)  Adnexal: (+)  Ducto sudoríparo | Granuloma: (0/+)  Macrophage(0/+)  Nerve: (-)  Vessels : (-)  Fibroblast: (-)  Pili muscle: (-)  Epidermis: (-)  Adnexal: (+)  Ducto sudoríparo | Granuloma: (0/+)  Macrophage(0/+)  Nerve: (-)  Vessels : (-)  Fibroblast: (-)  Pili muscle: (-)  Epidermis: (-)  Adnexal: (+)  Ducto sudoríparo | Granuloma: (0/+)  Macrophage(0/+)  Nerve: (-)  Vessels : (-)  Fibroblast: (-)  Pili muscle: (-)  Epidermis: (-)  Adnexal: (+)  Ducto sudoríparo | Granuloma: (+)  Macrophage(+)  Nerve: (-)  Vessels : (-)  Fibroblast: (-)  Pili muscle: (-)  Epidermis: (-)  Adnexal: (+)  Ducto sudoríparo | Granuloma: (0/+)  Macrophage(0/+)  Nerve: (-)  Vessels : (-)  Fibroblast: (-)  Pili muscle: (-)  Epidermis: (-)  Adnexal: (+)  Ducto sudoríparo | Granuloma: (0/+)  Macrophage(0/+)  Nerve: (-)  Vessels : (-)  Fibroblast: (-)  Pili muscle: (-)  Epidermis: (-)  Adnexal: (+)  Ducto sudoríparo |

**ANGPTL4 (angiopoietin-like 4), BAI1 (brain-specific angiogenesis inhibitor 1), BCAT1 (branched chain amino-acid transaminase 1, cytosolic), CD2 (CD2 molecule), CD27 (CD27 molecule), CD52 (CD52 molecule), EML2 (echinoderm microtubule associated protein like 2), FA2H (fatty acid 2-hydroxylase), GZMB (granzyme B), LIPA (Lysosomal Acid Lipase A), MMP9(matrix metallopeptidase 9), NCF1(neutrophil cytosolic factor 1), PTX3(pentraxin 3, long), SIGLEC15(sialic acid binding Ig-like lectin 15) and UBD(ubiquitin D).**

**CC: healthy controls**

**TT: tuberculoid**

**BT: borderline-tuberculoid**

**BB: borderline-borderline**

**BL: borderline-lepromatous**

**LL: lepromatous**

**R1: reaction type "1"**

**R2: Reaction type "2"**
