# Supplementary material for: Genome-Wide Screening of mRNA Expression in Leprosy Patients
Source: Front Genet. 2015 Nov 20;6:334. doi: 10.3389/fgene.2015.00334 (PMC4653304; doi:10.3389/fgene.2015.00334)
Supplement: Supplementary file 21 [file DataSheet15.DOCX]

**Suplement 21- References for the validation subject mRNAs by RT-PCR, associated with diseases or pathological processes signaling pathways.**

**AADACL3 (arylacetamidedeacetylase-like 3)**

**25-** Gregory SG, Barlow KF, McLay KE, Kaul R, Swarbreck D, Dunham A, et al.The DNA sequence and biological annotation of human chromosome 1.*Nature*(2006) **441(7091)**:315-21.doi:10.1038/nature04727.

**ACOT1 (acyl-CoA thioesterase 1)**

**26-** Yang S, Chen C, Wang H, Rao X, Wang F, Duan Q, et al.[Protective effects of Acyl-coAthioesterase 1 on diabetic heart via PPARα/PGC1α signaling.](http://www.ncbi.nlm.nih.gov/pubmed/23226270)*PLoS One.*(2012) **7(11)**:e50376.doi: 10.1371/journal.pone.0050376.

**ADAMTS4 (ADAM metallopeptidase with thrombospondin type 1 motif, 4)**

**27-** Yatabe T, Mochizuki S, Takizawa M, Chijiiwa M, Okada A, Kimura T, et al.[Hyaluronan inhibits expression of ADAMTS4 (aggrecanase-1) in human osteoarthritic chondrocytes.](http://www.ncbi.nlm.nih.gov/pubmed/18662930)*Ann Rheum Dis*(2009) **68(6)**:1051-8. doi: 10.1136/ard.2007.086884.

**28-** Minobe K, Ono R, Matsumine A, Shibata-Minoshima F, Izawa K, Oki T, et al. [Expression of ADAMTS4 in Ewing's sarcoma.](http://www.ncbi.nlm.nih.gov/pubmed/20664926)*Int J Oncol* (2010) **37(3)**:569-81.doi: 10.3892/ijo_00000706.

**AKR1B10 (aldo-ketoreductase family 1, member B10 (aldose reductase))**

**29-** Matsunaga T, Wada Y, Endo S, Soda M, El-Kabbani O, Hara A. [Aldo-KetoReductase 1B10 and Its Role in Proliferation Capacity of Drug-Resistant Cancers.](http://www.ncbi.nlm.nih.gov/pubmed/22319498)*Front Pharmacol*(2012) **31(3)**:5.doi: 10.3389/fphar.2012.00005.

**30-** Laffin B, Petrash JM. [Expression of the Aldo-Ketoreductases AKR1B1 and AKR1B10 in Human Cancers.](http://www.ncbi.nlm.nih.gov/pubmed/22685431)*Front Pharmacol*(2012)**6(3)**:104. doi: 10.3389/fphar.2012.00104.

**ALOX15B (arachidonate 15-lipoxygenase, type B)**

**31-** Magnusson LU, Lundqvist A, Karlsson MN, Skålén K, Levin M, Wiklund O, et al.[Arachidonate 15-lipoxygenase type B knockdown leads to reduced lipid accumulation and inflammation in atherosclerosis.](http://www.ncbi.nlm.nih.gov/pubmed/22912809)*PLoS One*(2012) **7(8)**:e43142. doi: 10.1371/journal.pone.0043142.

**32-** Daurkin I, Eruslanov E, Stoffs T, Perrin GQ, Algood C, Gilbert SM, et al. [Tumor-associated macrophages mediate immunosuppression in the renal cancer microenvironment by activating the 15-lipoxygenase-2 pathway.](http://www.ncbi.nlm.nih.gov/pubmed/21900394)*Cancer Res*(2011) **71(20)**:6400-9. doi: 10.1158/0008-5472.CAN-11-1261.

**33-**  Rydberg EK, Krettek A, Ullström C, Ekström K, Svensson PA, Carlsson LM, et al.[Hypoxia increases LDL oxidation and expression of 15 lipoxygenase-2 in human macrophages.](http://www.ncbi.nlm.nih.gov/pubmed/15358603)*ArteriosclerThr*ombVascBiol (2004) 24(11):2040-5. doi: 10.1161/01.atv.0000144951.08072.0b

**34-** Wuest SJ, Crucet M, Gemperle C, Loretz C, Hersberger M. [Expression and regulation of 12/15-lipoxygenases in human primary macrophages.](http://www.ncbi.nlm.nih.gov/pubmed/22980500) *Atherosclerosis*(2012) **225(1)**:121-7. doi: 10.1016/j.atherosclerosis.2012.07.022.

**35-** Danielsson KN, Rydberg EK, Ingelsten M, Akyürek LM, Jirholt P, Ullström C, et al. [15-Lipoxygenase-2 expression in human macrophages induces chemokine secretion and T cell migration.](http://www.ncbi.nlm.nih.gov/pubmed/18067895)Atherosclerosis(2008) 199(1):34-40. doi: 10.1016/j.atherosclerosis.2007.10.027.

**ANGPTL4 (angiopoietin-like 4)**

**36-** Tan MJ, Teo Z, Sng MK, Zhu P, Tan NS. [Emerging roles of angiopoietin-like 4 in human cancer.](http://www.ncbi.nlm.nih.gov/pubmed/22661548)*Mol Cancer Res*(2012) 10(6):677-88. doi: 10.1158/1541-7786.mcr-11-0519.

**37-** Guo L, Li SY, Ji FY, Zhao YF, Zhong Y, Lv XJ, et al. [Role of Angptl4 in vascular permeability and inflammation.](http://www.ncbi.nlm.nih.gov/pubmed/24173241)Inflamm Res(2014) 63(1):13-22. doi: 10.1007/s00011-013-0678-0

**38-**  Wang Y, Chen H, Li H, Zhang J, Gao Y. [Effect of angiopoietin-like protein 4 on rat pulmonary microvascular endothelial cells exposed to LPS.](http://www.ncbi.nlm.nih.gov/pubmed/23783408)*Int J Mol Med*(2013) **32(3)**:568-76. doi:10.3892/ijmm.2013.1420.

**39-** Hu J, Jham BC, Ma T, Friedman ER, Ferreira L, Wright JM, et al. [Angiopoietin-like4: a novel molecular hallmark in oral Kaposi's sarcoma.](http://www.ncbi.nlm.nih.gov/pubmed/21421336)*Oral Oncol*(2011) **47(5)**:371-5. doi: 10.1016/j.oraloncology.2011.02.018.

**AQP3 (aquaporin 3 (Gill blood group))**

**40-**Verkman AS. [Knock-out models reveal new aquaporin functions.](http://www.ncbi.nlm.nih.gov/pubmed/19096787)*HandbExpPharmacol* (2009) **190**:359-81.doi: 10.1007/978-3-540-79885-9_18.

**41-** Hara-Chikuma M, Verkman AS. [Roles of aquaporin-3 in the epidermis.](http://www.ncbi.nlm.nih.gov/pubmed/18548108)*J Invest Dermatol*(2008)**128(9)**:2145-51. doi: 10.1038/jid.2008.70.

**BAI1 (brain-specific angiogenesis inhibitor 1)**

**42-** Wang W, DA R, Wang M, Wang T, Qi L, Jiang H, et al. [Expression of brain-specific angiogenesis inhibitor 1 is inversely correlated with pathological grade, angiogenesis and peritumoral brain edema in human astrocytomas.](http://www.ncbi.nlm.nih.gov/pubmed/23761815)*OncolLett*(2013) **5(5)**:1513-1518. doi: 10.3892/ol.2013.1250.

**43-** Cork SM, Van Meir EG. [Emerging roles for the BAI1 protein family in the regulation of phagocytosis, synaptogenesis, neurovasculature, and tumor development.](http://www.ncbi.nlm.nih.gov/pubmed/21509575)*J Mol Med (Berl)*(2011) **89(8):**743-52. doi: 10.1007/s00109-011-0759-x.

**44-** Das S, Owen KA, Ly KT, Park D, Black SG, Wilson JM, et al. [Brain angiogenesis inhibitor 1 (BAI1) is a pattern recognition receptor that mediates macrophage binding and engulfment of Gram-negative bacteria.](http://www.ncbi.nlm.nih.gov/pubmed/21245295)*ProcNatlAcadSci U S A*(2011) **108(5)**:2136-41. doi: 10.1073/pnas.1014775108.

**45-** Wang W, DA R, Wang M, Wang T, Qi L, Jiang H, et al. [Expression of brain-specific angiogenesis inhibitor 1 is inversely correlated with pathological grade, angiogenesis and peritumoral brain edema in human astrocytomas.](http://www.ncbi.nlm.nih.gov/pubmed/23761815)*OncolLett*(2013) **5(5)**:1513-1518.doi: 10.3892/ol.2013.1250.

**BCAT1 (branched chain amino-acid transaminase 1, cytosolic)**

**46-**Tönjes M, Barbus S, Park YJ, Wang W, Schlotter M, Lindroth AM, et al. [BCAT1 promotes cell proliferation through amino acid catabolism in gliomas carrying wild-type IDH1.](http://www.ncbi.nlm.nih.gov/pubmed/23793099)*Nat Med*(2013) **19(7)**:901-8. doi: 10.1038/nm.3217.

**47-** Zhou W, Feng X, Ren C, Jiang X, Liu W, Huang W, et al. [Over-expression of **BCAT1**, a c-Myc target gene, induces cell proliferation, migration and invasion in nasopharyngeal carcinoma.](http://www.ncbi.nlm.nih.gov/pubmed/23758864)*Mol Cancer*(2013) **8(12)**:53. doi: 10.1186/1476-4598-12-53.

**48-** de Bont JM, Kros JM, Passier MM, Reddingius RE, SillevisSmitt PA, Luider TM, et al. [Differential expression and prognostic significance of SOX genes in pediatric medulloblastoma and ependymoma identified by microarray analysis.](http://www.ncbi.nlm.nih.gov/pubmed/18577562)*NeuroOncol*(2008) **10(5)**:648-60. doi: 10.1215/15228517-2008-032.

**CAV2 (caveolin 2)**

**49-** Lee S, Kwon H, Jeong K, Pak Y. [Regulation of cancer cell proliferation by caveolin-2 down-regulation and re-expression.](http://www.ncbi.nlm.nih.gov/pubmed/21373752)*Int J Oncol*(2011) **38(5)**:1395-402. doi: 10.3892/ijo.2011.958. doi: 10.3892/ijo.2011.958.

**C1orf93 (FAM213B (C1ORF93) family with sequence similarity 213, member B)**

**50-** Anderson CA, Boucher G, Lees CW, Franke A, D'Amato M, Taylor KD, et al. [Meta-analysis identifies 29 additional ulcerative colitis risk loci, increasing the number of confirmed associations to 47.](http://www.ncbi.nlm.nih.gov/pubmed/21297633)*Nat Genet*(2011) **43(3)**:246-52. doi: 10.1038/ng.764.

**C3AR1 (complement component 3a receptor 1)**

**51-** Mizuno M, Blanchin S, Gasque P, Nishikawa K, Matsuo S. [High levels of complement C3a receptor in the glomeruli in lupus nephritis.](http://www.ncbi.nlm.nih.gov/pubmed/17472841)*Am J Kidney Dis*(2007) **49(5)**:598-606.doi: 10.1053/j.ajkd.2007.02.271.

**CD2 (CD2 molecule)**

**52-** Li J, Qi B, Chen P, He L, Wang P, Ji Y, et al. [The expression of CD2 in chronic HBV infection.](http://www.ncbi.nlm.nih.gov/pubmed/18318997)*Cell MolImmunol*(2008) **5(1)**:69-73. doi: 10.1038/cmi.2008.9.

**53-** Sullivan L, Sano S, Pirmez C, Salgame P, Mueller C, Hofman F, et al.[Expression of adhesionmolecules in leprosylesions.](http://www.ncbi.nlm.nih.gov/pubmed/1718871)*Infect Immun*(1991) **59(11)**:4154-60.

**54-** Kalland ME, Oberprieler NG, Vang**T**, Taskén K, Torgersen KM. [Tcell-signalingnetworkanalysis reveals distinct differences between CD28 and CD2 costimulation responses in various subsets and in the MAPK pathway between resting and activated regulatory T cells.](http://www.ncbi.nlm.nih.gov/pubmed/22013130)*J Immunol*(2011) **187(10)**:5233-45.doi: 10.4049/jimmunol.1101804.

**55-** Lorvik KB, Haabeth OA, Clancy T, Bogen B, Corthay A. [Molecular profiling of tumor-specific T_H_1 cells activated in vivo.](http://www.ncbi.nlm.nih.gov/pubmed/23762808)*Oncoimmunology* (2013) **2(5)**:e24383.doi: 10.4161/onci.24383.

**CD3D (CD3dmolecule, delta (CD3-TCR complex))**

**56-** West NR, Milne K, Truong PT, Macpherson N, Nelson BH, Watson PH.

[Tumor-infiltrating lymphocytes predict response to anthracycline-based chemotherapy in estrogen receptor-negative breast cancer.](http://www.ncbi.nlm.nih.gov/pubmed/22151962)*Breast Cancer Res*(2011) **13(6)**:R126. doi: 10.1186/bcr3072.

**CD3G (CD3g molecule, gamma (CD3-TCR complex))**

**57-** Rutledge WC, Kong J, Gao J, Gutman DA, Cooper LA, Appin C, et al. [Tumor-infiltrating lymphocytes in glioblastoma are associated with specific genomic alterations and related to transcriptional class.](http://www.ncbi.nlm.nih.gov/pubmed/23864165)*Clin Cancer Res*(2013) **19(18)**:4951-60. doi: 10.1158/1078-0432.ccr-13-0551.

**CD27 (CD27 molecule)**

**58-** Nolte MA, van Olffen RW, van Gisbergen KP, van Lier RA. [Timing and tuning ofCD27-CD70 interactions: the impact of signal strength in setting the balance between adaptive responses and immunopathology.](http://www.ncbi.nlm.nih.gov/pubmed/19426224)*ImmunolRev* (2009) **229(1)**:216-31. doi: 10.1111/j.1600-065x.2009.00774.x.

**CD48 (CD48 molecule)**

**59-** Hosen N, Ichihara H, Mugitani A, Aoyama Y, Fukuda Y, Kishida S, et al. [CD48 as a novel molecular target for antibody therapy in multiple myeloma.](http://www.ncbi.nlm.nih.gov/pubmed/22098460)*Br J Haematol*(2012) **56(2)**:213-24. doi: 10.1111/j.1365-2141.2011.08941.x.

**CD52 (CD52 molecule)**

**60-** Freedman MS, Kaplan JM, Markovic-Plese S. [Insights into the Mechanisms of the Therapeutic Efficacy of Alemtuzumab in Multiple Sclerosis.](http://www.ncbi.nlm.nih.gov/pubmed/24363961)*J Clin Cell Immunol*(2013) 4(4): pii1000152. doi: 10.4172/2155-9899.1000152.

**61-** Santos DD, Hatjiharissi E, Tournilhac O, Chemaly MZ, Leleu X, Xu L, et al. [CD52 is expressed on human mast cells and is a potential therapeutic target in Waldenstrom'sMacroglobulinemia and mast cell disorders.](http://www.ncbi.nlm.nih.gov/pubmed/16796779)*Clin Lymphoma Myeloma* (2006) **6(6)**:478-83.doi: 10.3816/clm.2006.n.029.

**62-** Samten B. [CD52 as both a marker and an effector molecule of T cells with regulatory action: Identification of novel regulatory T cells.](http://www.ncbi.nlm.nih.gov/pubmed/24037183)*Cell MolImmunol*(2013) **10(6)**:456-8. doi: 10.1038/cmi.2013.38.

**63-** Klotz L, Wiendl H. [Monoclonal antibodies in neuroinflammatory diseases.](http://www.ncbi.nlm.nih.gov/pubmed/23521026)

*Expert OpinBiolTher*(2013) **13(6)**:831-46.doi: 10.1517/14712598.2013.767329.

**CD53 (CD53 molecule)**

**64-** Pedersen-Lane JH, Zurier RB, Lawrence DA.[Analysis of the thiol status of peripheral blood leukocytes in rheumatoid arthritis patients.](http://www.ncbi.nlm.nih.gov/pubmed/17210617)*J LeukocBiol*(2007) **81(4)**:934-41. doi: 10.1189/jlb.0806533.

**CD300E (CD300e molecule)**

**65-** Gasiorowski RE, Ju X, Hart DN, Clark GJ. [CD300 molecule regulation of human dendritic cell functions.](http://www.ncbi.nlm.nih.gov/pubmed/23072861)*ImmunolLett*(2013) **149(1-2)**:93-100. doi: 10.1016/j.imlet.2012.10.005.

**CHIT1 (chitinase 1 (chitotriosidase))**

**66-** Kanneganti M, Kamba A, Mizoguchi E. [Role of chitotriosidase (chitinase 1) under normal and disease conditions.](http://www.ncbi.nlm.nih.gov/pubmed/23439988)*J EpithelBiolPharmacol*(2012) **5**:1-9.doi: 10.1016/j.imlet.2012.10.005.

**CIDEC (cell death-inducing DFFA-like effector c)**

**67-** Jinno Y, Nakakuki M, Sato A, Kawano H, Notsu T, Mizuguchi K, et al. [Cide-a andCide-c are induced in the progression of hepatic steatosis and inhibited by eicosapentaenoic acid.](http://www.ncbi.nlm.nih.gov/pubmed/20542418)*Prostaglandins LeukotEssent Fatty Acids*(2010) **83(2)**:75-81.doi: 10.1016/j.plefa.2010.05.002.

**CRABP1 (cellular retinoic acid binding protein 1)**

**68-** Miyake T, Ueda Y, Matsuzaki S, Miyatake T, Yoshino K, Fujita M, et al. [CRABP1-reduced expression is associated with poorer prognosis in serous and clear cell ovarian adenocarcinoma.](http://www.ncbi.nlm.nih.gov/pubmed/20571827)*J Cancer Res ClinOncol* (2011) **137(4)**:715-22. doi: 10.1007/s00432-010-0930-8.

**CRAT (carnitine O-acetyltransferase)**

**69-** Seiler SE, Martin OJ, Noland RC, Slentz DH, Debalsi KL, Ilkayeva OR, et al. [Obesity and Lipid Stress Inhibit CarnitineAcetyltransferase Activity.](http://www.ncbi.nlm.nih.gov/pubmed/24395925)*J Lipid Res*(2014) **55(4)**:635-44.doi: 10.1194/jlr.m043448.

**CTSZ (cathepsin Z)**

**70-** Wang J, Chen L, Li Y, Guan XY. [Overexpression of cathepsin Z contributes to tumor metastasis by inducing epithelial-mesenchymal transition in hepatocellular carcinoma.](http://www.ncbi.nlm.nih.gov/pubmed/21966391)*PLoS One*(2011) **6(9)**:e24967. doi: 10.1371/journal.pone.0024967.

**71-** Lines KE, Chelala C, Dmitrovic B, Wijesuriya N, Kocher HM, Marshall JF, et al. [S100P-binding protein, S100PBP, mediates adhesion through regulation of cathepsin Z in pancreatic cancer cells.](http://www.ncbi.nlm.nih.gov/pubmed/22330678)*Am J Pathol*(2012) **180(4)**:1485-94. doi: 10.1016/j.ajpath.2011.12.031.

**CXorf65 (chromosome X open reading frame 65)**

**72-** Ross MT, Grafham DV, Coffey AJ, Scherer S, McLay K, Muzny D, et al. The DNA sequence of the human X chromosome.*Nature*(2005) **434(7031)**:325-37.doi: 10.1038/nature03440.

**CYBB (cytochrome b-245, beta polypeptide)**

**73-** Schiavone S, Jaquet V, Sorce S, Dubois-Dauphin M, Hultqvist M, Bäckdahl L, et al. [NADPH oxidase elevations in pyramidal neurons drive psychosocial stress-induced neuropathology.](http://www.ncbi.nlm.nih.gov/pubmed/22832955)*TranslPsychiatry* (2012) **8(2)**:e111. doi: 10.1038/tp.2012.36.

**74-** Djamali A, Reese S, Hafez O, Vidyasagar A, Jacobson L, Swain W, et al. [Nox2 is a mediator of chronic CsA nephrotoxicity.](http://www.ncbi.nlm.nih.gov/pubmed/22568654)*Am J Transplant*(2012) **12(8)**:1997-2007. doi: 10.1111/j.1600-6143.2012.04081.x.

**EML2 (echinoderm microtubule associated protein like 2)**

**75-** Duong CV, Emes RD, Wessely F, Yacqub-Usman K, Clayton RN, Farrell WE. [Quantitative, genome-wide analysis of the DNA methylome in sporadic pituitary adenomas.](http://www.ncbi.nlm.nih.gov/pubmed/23045325)*EndocrRelat Cancer*(2012) **19(6)**:805-16. doi: 10.1530/erc-12-0251.

**76-** Liu D, Yao S, Wise GE. [Regulation of SFRP-1 expression in the rat dental follicle.](http://www.ncbi.nlm.nih.gov/pubmed/22313323)*Connect Tissue Res*(2012) **53(5)**:366-72. doi: 10.3109/03008207.2012.664204.

**FA2H (fatty acid 2-hydroxylase)**

**77-** Kruer MC, Gregory A, Hayflick SJ. “[Fatty Acid Hydroxylase-Associated Neurodegeneration”.](http://www.ncbi.nlm.nih.gov/pubmed/21735565)In: Pagon RA, Adam MP, Bird TD, Dolan CR, Fong CT, Stephens K, editors. GeneReviews™ [Internet]. Seattle, WA: University of Washington, Seattle; 1993-2015. 2011 Jun 28 [updated 2012 Sep 20].

**78-** Schneider SA, Dusek P, Hardy J, Westenberger A, Jankovic J, Bhatia KP. [Genetics and Pathophysiology of Neurodegeneration with Brain Iron Accumulation (NBIA).](http://www.ncbi.nlm.nih.gov/pubmed/23814539)*CurrNeuropharmacol*(2013) **11(1)**:59-79.doi: 10.2174/157015913804999469.

**79-** Maier H, Meixner M, Hartmann D, Sandhoff R, Wang-Eckhardt L, Zöller I, et al. [Normal fur development and sebum production depends on fatty acid 2-hydroxylase expression in sebaceous glands.](http://www.ncbi.nlm.nih.gov/pubmed/21628453)*J BiolChem*(2011) **286(29)**:25922-34. doi: 10.1074/jbc.m111.231977.

**80-** Potter KA, Kern MJ, Fullbright G, Bielawski J, Scherer SS, Yum SW, et al. [Central nervous system dysfunction in a mouse model ofFA2H deficiency.](http://www.ncbi.nlm.nih.gov/pubmed/21491498)*Glia*(2011) **59(7)**:1009-21. doi: 10.1002/glia.21172.

**FADS2 (FADS2 fatty acid desaturase 2)**

**81-** Merino DM, Ma DW, Mutch DM. [Genetic variation in lipiddesaturases and its impact on the development of human disease.](http://www.ncbi.nlm.nih.gov/pubmed/20565855)*Lipids Health Dis*(2010) **9**:63. doi: 10.1186/1476-511x-9-63.

**FAM180B (family with sequence similarity 180, member B)**

**82-** Strausberg RL, Feingold EA, Grouse LH, Derge JG, Klausner RD, Collins FS, et al. Mammalian Gene Collection Program Team.Generation and initial analysis of more than 15,000 full-length human and mouse cDNA sequences.*ProcNatlAcadSci U S A* (2002) **99(26)**:16899-903. doi: 10.1073/pnas.242603899.

**FAM26F (Family With Sequence Similarity 26, Member F)**

**83-** Julià A, Domènech E, Chaparro M, García-Sánchez V, Gomollón F, Panés J, et al. A genome-wide association study identifies a novel locus at 6q22.1 associated with ulcerative colitis.*Hum Mol Genet*(2014) **23(25)**:6927-34.doi: 10.1093/hmg/ddu398.

**FASN (fatty acid synthase)**

**84-** Wei X, Yang Z, Rey FE, Ridaura VK, Davidson NO, Gordon JI, et al. [Fatty acid synthase modulates intestinal barrier function through palmitoylation of mucin 2.](http://www.ncbi.nlm.nih.gov/pubmed/22341463)*Cell Host Microbe*(2012) **11(2)**:140-52. doi: 10.1016/j.chom.2011.12.006.

**85-** Wu X, Qin L, Fako V, Zhang JT. [Molecular mechanisms of fatty acid synthase (FASN)-mediated resistance to anti-cancer treatments.](http://www.ncbi.nlm.nih.gov/pubmed/24080588)*AdvBiolRegul* (2014) **54**:214-21.doi: 10.1016/j.jbior.2013.09.004.

**FGFBP1 (fibroblast growth factor binding protein 1)**

**86-** Schulze D, Plohmann P, Höbel S, Aigner A. [Anti-tumor effects of fibroblast growth factor-binding protein (FGF-BP) knockdown in colon carcinoma.](http://www.ncbi.nlm.nih.gov/pubmed/22111880)*Mol Cancer*(2011) **10**:144. doi: 10.1186/1476-4598-10-144.

**87-** Werner S. [A novel enhancer of the wound healing process: the fibroblast growth factor-binding protein.](http://www.ncbi.nlm.nih.gov/pubmed/21964189)*Am J Pathol*(2011) **179(5)**:2144-7. doi: 10.1016/j.ajpath.2011.09.001.

**88-** Tassi E, McDonnell K, Gibby KA, Tilan JU, Kim SE, Kodack DP, et al. [Impact of fibroblast growth factor-binding protein-1 expression on angiogenesis and wound healing.](http://www.ncbi.nlm.nih.gov/pubmed/21945411)*Am J Pathol*(2011) **179(5)**:2220-32. doi: 10.1016/j.ajpath.2011.07.043.

**FOXQ1 (forkhead box Q1)**

**89-** Christensen J, Bentz S, Sengstag T, Shastri VP, Anderle P. [FOXQ1, a novel target of the Wnt pathway and a new marker for activation of Wnt signaling in solid tumors.](http://www.ncbi.nlm.nih.gov/pubmed/23555880)*PLoS One* (2013) **8(3)**:e60051. doi: 10.1371/journal.pone.0060051.

**FYB (FYN binding protein)**

**90-** Addobbati C, Brandão LA, Guimarães RL, Pancotto JA, Donadi EA, Crovella S, et al. [**FYB** gene polymorphisms are associated with susceptibility for systemic lupus erythemathosus (SLE).](http://www.ncbi.nlm.nih.gov/pubmed/23628395)*Hum Immunol* (2013) **74(8)**:1009-14. doi: 10.1016/j.humimm.2013.04.026.

**GAL (galanin)**

**91-** Counts SE, Perez SE, Ginsberg SD, Mufson EJ.[Neuroprotective role for galaninin Alzheimer's disease.](http://www.ncbi.nlm.nih.gov/pubmed/21299067)*EXS*(2010) **102**:143-62.doi: 10.1007/978-3-0346-0228-0_11.

**92-** Webling KE, Runesson J, Bartfai T, Langel U. [Galanin receptors and ligands.](http://www.ncbi.nlm.nih.gov/pubmed/23233848)*Front Endocrinol* (2012) 3:146. doi: 10.3389/fendo.2012.00146.

**93-** Misawa K, Kanazawa T, Misawa Y, Uehara T, Imai A, Takahashi G, et al. [Galanin has tumor suppressor activity and is frequently inactivated by aberrant promoter methylation in head and neck cancer.](http://www.ncbi.nlm.nih.gov/pubmed/23730414)*TranslOncol* (2013) **6(3)**:338-46.doi: 10.1593/tlo.13115.

**94-** Wraith DC, Pope R, Butzkueven H, Holder H, Vanderplank P, Lowrey P, et al. [A role for galanin in human and experimental inflammatory demyelination.](http://www.ncbi.nlm.nih.gov/pubmed/19717462)*ProcNatlAcadSci U S A*(2009) **106(36)**:15466-71. doi:10.1073/pnas.0903360106.

**GBP1(guanylate binding protein 1, interferon-inducible)**

**95-** Selleck EM, Fentress SJ, Beatty WL, Degrandi D, Pfeffer K, Virgin HW 4th, et al. [Guanylate-binding protein 1 (**Gbp1**) contributes to cell-autonomous immunity against Toxoplasma gondii.](http://www.ncbi.nlm.nih.gov/pubmed/23633952)*PLoSPathog*(2013) **9(4)**:e1003320. doi: 10.1371/journal.ppat.1003320.

**96-** Pan W, Zuo X, Feng T, Shi X, Dai J. [Guanylate-binding protein 1 participates in cellular antiviral response to dengue virus.](http://www.ncbi.nlm.nih.gov/pubmed/23186538)*Virol J*(2012) **9**:292. doi: 10.1186/1743-422x-9-292.

**GJB2 (gap junction protein, beta 2, 26kDa)**

**97-** Lee JR, White TW. [Connexin-26 mutations in deafness and skin disease.](http://www.ncbi.nlm.nih.gov/pubmed/19939300)*Expert Rev Mol Med*(2009) **11**:e35. doi: 10.1017/s1462399409001276.

**GPNMB (glycoprotein (transmembrane) nmb)**

**98-** Maric G, Rose AA, Annis MG, Siegel PM. [Glycoprotein non-metastatic b (GPNMB): A metastatic mediator and emerging therapeutic target in cancer.](http://www.ncbi.nlm.nih.gov/pubmed/23874106)*Onco Targets Ther*(2013) **6**:839-52.doi: 10.2147/ott.s44906.

**IRF8 (interferon regulatory factor 8)**

**GZMB (granzyme B (granzyme 2, cytotoxic T-lymphocyte-associated serine esterase 1))**

**99-** Abdou AG, Shoeib M, Bakry OA, El-Bality H. [Immunohistochemical expression of granzyme B and perforin in discoid lupus erythematosus.](http://www.ncbi.nlm.nih.gov/pubmed/23980805)*UltrastructPathol(*2013) **37(6)**:408-16. doi: 10.3109/01913123.2013.816400.

**100-** Darrah E, Rosen A. [Granzyme B cleavage of autoantigens in autoimmunity.](http://www.ncbi.nlm.nih.gov/pubmed/20075942)*Cell Death Differ*(2010) **17(4)**:624-32. doi: 10.1038/cdd.2009.197.

**101-** Marcet-Palacios M, Duggan BL, Shostak I, Barry M, Geskes T, Wilkins JA, et al. [Granzyme B inhibits vaccinia virus production through proteolytic cleavage of eukaryotic initiation factor 4 gamma 3.](http://www.ncbi.nlm.nih.gov/pubmed/22194691)

*PLoSPathog*(2011) **7(12)**:e1002447. doi: 10.1371/journal.ppat.1002447.

**HMGCS1 (3-hydroxy-3-methylglutaryl-CoA synthase 1 (soluble))**

**102-** Mirza Z, Kamal MA, Abuzenadah AM, Al-Qahtani MH, Karim S. [Establishing Genomic/Transcriptomic Links between Alzheimer's Disease and Type II Diabetes Mellitus by Meta-Analysis Approach.](http://www.ncbi.nlm.nih.gov/pubmed/24059308)*CNS NeurolDisord Drug Targets*(2013) **13(3)**:501-16.doi: 10.2174/18715273113126660154.

**IGJ (immunoglobulin J polypeptide, linker protein for immunoglobulin alpha and mu polypeptides)**

**103-** Streicher K, Morehouse CA, Groves CJ, Rajan B, Pilataxi F, Lehmann KP, et al. [The plasma cell signature in autoimmune disease.](http://www.ncbi.nlm.nih.gov/pubmed/24431284)*Arthritis Rheumatol*(2014) **66(1)**:173-84. doi: 10.1002/art.38194.

**IGLL1 (immunoglobulin lambda-like polypeptide 1)**

**,104-** Hu K, Chen F. [Identification of significant pathways in gastric cancer based on protein-protein interaction networks and cluster analysis.](http://www.ncbi.nlm.nih.gov/pubmed/23055812)*Genet MolBiol*(2012) **35(3)**:701-8. doi: 10.1590/s1415-47572012005000045.

**IGLL5 (immunoglobulin lambda-like polypeptide 5)**

**105-** Akiyama M, Yamada O, Agawa M, Yuza Y, Yanagisawa T, Eto Y, et al. [Effects of prednisolone on specifically expressed **genes** in pediatric acute B-lymphoblastic leukemia.](http://www.ncbi.nlm.nih.gov/pubmed/18391702)*J PediatrHematolOncol* (2008) **30(4)**:313-6. doi: 10.1097/mph.0b013e318161a28f.

**IL1B (interleukin1, beta)**

**106-** Novikov A, Cardone M, Thompson R, Shenderov K, Kirschman KD, Mayer-Barber KD, et al. [Mycobacterium tuberculosis triggers host type I IFN signaling to regulate IL-1β production in human macrophages.](http://www.ncbi.nlm.nih.gov/pubmed/21784976)*J Immunol*(2011) **187(5)**:2540-7. doi: 10.4049/jimmunol.1100926.

**107-** Xu J, Yin Z, Cao S, Gao W, Liu L, Yin Y, et al. [Systematic review and meta-analysis on the association between IL-1B polymorphisms and cancer risk.](http://www.ncbi.nlm.nih.gov/pubmed/23704929)*PLoS One*(2013) **8(5)**:e63654. doi: 10.1371/journal.pone.0063654.

**IL7R (interleukin 7 receptor)**

**108-** Olsson T, Jagodic M, Piehl F, Wallström E. [Genetics of autoimmune neuroinflammation.](http://www.ncbi.nlm.nih.gov/pubmed/16973343)*CurrOpinImmunol*(2006) **18(6)**:643-9.doi: 10.1016/j.coi.2006.08.001.

**109-** Heron M, Grutters JC, van Moorsel CH, Ruven HJ, Huizinga TW, van der Helm-van Mil AH, et al. [Variation in IL7Rpredisposes to sarcoid inflammation.](http://www.ncbi.nlm.nih.gov/pubmed/19626041)*Genes Immun*(2009) **10(7)**:647-53. doi: 10.1038/gene.2009.55.

**IL17RA (interleukin 17 receptor A)**

**110-** Raychaudhuri SP. [Role of IL-17 in psoriasis and psoriatic arthritis.](http://www.ncbi.nlm.nih.gov/pubmed/22362575)*Clin Rev Allergy Immunol*(2013) **44(2)**:183-93. doi: 10.1007/s12016-012-8307-1.

**111-** Berghout J, Langlais D, Radovanovic I, Tam M, MacMicking JD, Stevenson MM, et al. [**Irf8**-regulated genomic responses drive pathological inflammation during cerebral malaria.](http://www.ncbi.nlm.nih.gov/pubmed/23853600)*PLoSPathog*(2013) **9(7)**:e1003491. doi: 10.1371/journal.ppat.1003491.

**112-** Salem S, Gros P. [Genetic determinants of susceptibility to Mycobacterial infections:IRF8, a new kid on the block.](http://www.ncbi.nlm.nih.gov/pubmed/23468103)*AdvExp Med Biol*(2013) **783**:45-80.doi: 10.1007/978-1-4614-6111-1_3.

**ITGAL (integrin, alpha L (antigen CD11A (p180), lymphocyte function-associated antigen 1; alpha polypeptide))**

**113-** Jeffries MA, Sawalha AH. [Epigenetics in systemic lupus erythematosus: leading the way for specific therapeutic agents.](http://www.ncbi.nlm.nih.gov/pubmed/22184503)*Int J ClinRheumtol*(2011) **6(4)**:423-439.doi: 10.2217/ijr.11.32.

**KLK6 (kallikrein-related peptidase 6)**

**114-** Pampalakis G, Prosnikli E, Agalioti T, Vlahou A, Zoumpourlis V, Sotiropoulou G. [A tumor-protective role for human kallikrein-related peptidase 6 in breast cancer mediated by inhibition of epithelial-to-mesenchymal transition.](http://www.ncbi.nlm.nih.gov/pubmed/19383923)*Cancer Res*(2009) **69(9)**:3779-87.doi: 10.1158/0008-5472.can-08-1976.

**115-** Yousef GM, Kishi T, Diamandis EP. [Role of kallikrein enzymes in the central nervous system.](http://www.ncbi.nlm.nih.gov/pubmed/12589961)*ClinChimActa*(2003) **329(1-2)**:1-8.doi: 10.1016/s0009-8981(03)00004-4.

**KRT6C (keratin 6C)**

**116-** Wilson NJ, Messenger AG, Leachman SA, O'Toole EA, Lane EB, McLean WH, Smith FJ. [Keratin K6c mutations cause focal palmoplantarkeratoderma.](http://www.ncbi.nlm.nih.gov/pubmed/19609311)*J Invest Dermatol*(2010) **130(2)**:425-9. doi: 10.1038/jid.2009.215.

**LCK (lymphocyte-specific protein tyrosine kinase)**

**117-** DiMauro EF, Newcomb J, Nunes JJ, Bemis JE, Boucher C, Chai L, et al. [Structure-guided design of aminopyrimidine amides as potent, selective inhibitors of lymphocyte specific kinase: synthesis, structure-activity relationships, and inhibition of in vivo T cell activation.](http://www.ncbi.nlm.nih.gov/pubmed/18321037)*J MedChem* (2008) **51(6)**:1681-94. doi: 10.1021/jm7010996.

**LDHD (lactatedehydrogenase D)**

**118-** de Bari L, Moro L, Passarella S. [Prostate cancer cells metabolize d-lactate inside mitochondria via a D-lactate dehydrogenase which is more active and highly expressed than in normal cells.](http://www.ncbi.nlm.nih.gov/pubmed/23333299)*FEBS Lett*(2013) **587(5)**:467-73. doi: 10.1016/j.febslet.2013.01.011.

**LGMN (legumain)**

**119-** Wang L, Chen S, Zhang M, Li N, Chen Y, Su W, et al. [Legumain: a biomarker for diagnosis and prognosis of human ovarian cancer.](http://www.ncbi.nlm.nih.gov/pubmed/22441772)*J Cell Biochem*(2012) **113(8)**:2679-86. doi: 10.1002/jcb.24143.

**LIPA (Lysosomalacid lipase A)**

**120-** Zschenker O, Illies T, Ameis D. [Overexpression of **lysosomal acid lipase** and other proteins in atherosclerosis.](http://www.ncbi.nlm.nih.gov/pubmed/16877765)*J Biochem*(2006) **140(1)**:23-38. doi: 10.1093/jb/mvj137.

**LTB (lymphotoxin beta (TNF superfamily, member 3))**

**121-** Young J, Yu X, Wolslegel K, Nguyen A, Kung C, Chiang E, et al. [Lymphotoxin-alphabetaheterotrimers are cleaved by metalloproteinases and contribute to synovitis in rheumatoid arthritis.](http://www.ncbi.nlm.nih.gov/pubmed/20356761)*Cytokine* (2010) **51(1)**:78-86.doi: 10.1016/j.cyto.2010.03.003.

**MGST1(microsomalglutathione S-transferase1)**

**122-** Maeda A, Crabb JW, Palczewski K. [Microsomal glutathione S-transferase 1 in the retinal pigment epithelium: protection against oxidative stress and a potential role in aging.](http://www.ncbi.nlm.nih.gov/pubmed/15641772)*Biochemistry*(2005) **44(2)**:480-9.doi: 10.1021/bi048016f.

**123-** Johansson K, Ito M, Schophuizen CM, Mathew Thengumtharayil S, Heuser VD, Zhang J, et al. [Characterization of new potential anticancer drugs designed to overcome glutathione transferase mediated resistance.](http://www.ncbi.nlm.nih.gov/pubmed/21851097)*Mol Pharm*(2011) **8(5)**:1698-708. doi: 10.1021/mp2000692.

**MICAL2 (microtubule associated monooxygenase, calponin and LIM domain containing 2)**

**124-** Ashida S, Furihata M, Katagiri T, Tamura K, Anazawa Y, Yoshioka H, et al. [Expression of novel molecules, MICAL2-PV (MICAL2 prostate cancer variants), increases with high Gleason score and prostate cancer progression.](http://www.ncbi.nlm.nih.gov/pubmed/16675569)*Clin Cancer Res*(2006) **12(9)**:2767-73.doi: 10.1158/1078-0432.ccr-05-1995.

**MMP9 (matrix metallopeptidase 9)**

**125-** Vandooren J, Van den Steen PE, Opdenakker G. [Biochemistry and molecular biology of gelatinase B or matrix metalloproteinase-9 (MMP-9): the next decade.](http://www.ncbi.nlm.nih.gov/pubmed/23547785)*Crit Rev BiochemMolBiol*(2013) **48(3)**:222-72. doi: 10.3109/10409238.2013.770819.

**126-** Campos AH, Vassallo J, Soares FA. [Matrix metalloproteinase-9 expression by hodgkin-reed-sternberg cells is associated with reduced overall survival in young adult patients with classical hodgkin lymphoma.](http://www.ncbi.nlm.nih.gov/pubmed/24086377)*PLoS One*(2013) **8(9)**:e74793.doi:10.1371/journal.pone.0074793.

**MT2A (metallothionein 2A)**

**127-** Arion D, Unger T, Lewis DA, Levitt P, Mirnics K. [Molecular evidence for increased expression of genes related to immune and chaperone function in the prefrontal cortex in schizophrenia.](http://www.ncbi.nlm.nih.gov/pubmed/17568569)*Biol Psychiatry* (2007) **62(7):**711-21. doi: 10.1016/j.biopsych.2006.12.021.

**128-** Werynska B, Pula B, Muszczynska-Bernhard B, Gomulkiewicz A, Piotrowska A, Prus R, et al. [Metallothionein 1F and 2A overexpression predicts poor outcome of non-small cell lung cancer patients.](http://www.ncbi.nlm.nih.gov/pubmed/23064051)*ExpMolPathol*(2013) **94(1)**:301-8. doi: 10.1016/j.yexmp.2012.10.006.

**129-** Haynes V, Connor T, Tchernof A, Vidal H, Dubois S. [Metallothionein 2a gene expression is increased in subcutaneous adipose tissue of type 2 diabetic patients.](http://www.ncbi.nlm.nih.gov/pubmed/23148893)*Mol Genet Metab*(2013) **108(1)**:90-4. doi: 10.1016/j.ymgme.2012.10.012.

**NCF1 (neutrophil cytosolic factor 1)**

**130-** Leiding JW, Holland SM. In: Pagon RA, Adam MP, Bird TD, Dolan CR, Fong CT, Stephens K, editors. GeneReviews™ [Internet]. Seattle (WA): University of Washington, Seattle; 1993-2013. 2012 Aug 09.[Chronic Granulomatous Disease.](http://www.ncbi.nlm.nih.gov/pubmed/22876374)

**131-** Gauss KA, Nelson-Overton LK, Siemsen DW, Gao Y, DeLeo FR, Quinn MT. [Role of NF-kappaB in transcriptional regulation of the phagocyte NADPH oxidase by tumor necrosis factor-alpha.](http://www.ncbi.nlm.nih.gov/pubmed/17537988)*J LeukocBiol*(2007) **82(3)**:729-41.doi:10.1189/jlb.1206735.

**132-** Efimova O, Szankasi P, Kelley TW.**[Ncf1](http://www.ncbi.nlm.nih.gov/pubmed/21253614)** [(p47phox) is essential for direct regulatory T cell mediated suppression of CD4+ effector T cells.](http://www.ncbi.nlm.nih.gov/pubmed/21253614)*PLoS One*(2011) **6(1)**:e16013. doi: 10.1371/journal.pone.0016013.

**NFKBIE (nuclear factor of kappa light polypeptide gene enhancer in B-cells inhibitor, epsilon)**

**133-** Myouzen K, Kochi Y, Okada Y, Terao C, Suzuki A, Ikari K, et al. [Functional variants in NFKBIE and RTKN2 involved in activation of the NF-κB pathway are associated with rheumatoid arthritis in Japanese.](http://www.ncbi.nlm.nih.gov/pubmed/23028356)*PLoS Genet*(2012)**8(9)**:e1002949. doi: 10.1371/journal.pgen.1002949.

**NGEF (neuronal guanine nucleotide exchange factor)**

**134-** Rosas OR, Figueroa JD, Torrado AI, Rivera M, Santiago JM, Konig-Toro F, et al. [Expression and activation of ephexin is altered after spinal cord injury.](http://www.ncbi.nlm.nih.gov/pubmed/20949525)

*DevNeurobiol*(2011) **71(7)**:595-607. doi: 10.1002/dneu.20848.

**NLRC3 (CARD domain containing 3)**

**135-** Schneider M, Zimmermann AG, Roberts RA, Zhang L, Swanson KV, Wen H, et al. [The innate immune sensor NLRC3 attenuates Toll-like receptor signaling via modification of the signaling adaptor TRAF6 and transcription factor NF-κB.](http://www.ncbi.nlm.nih.gov/pubmed/22863753)*Nat Immunol*(2012) **13(9)**:823-31. doi: 10.1038/ni.2378.

**NNAT (neuronatin)**

**136-** Revill K, Dudley KJ, Clayton RN, McNicol AM, Farrell WE. [Loss of neuronatin expression is associated with promoter hypermethylation in pituitary adenoma.](http://www.ncbi.nlm.nih.gov/pubmed/19218280)*EndocrRelat Cancer*(2009)**16(2)**:537-48.doi: 10.1677/erc-09-0008.

**137-** Vrang N, Meyre D, Froguel P, Jelsing J, Tang-Christensen M, Vatin V, et al. [The imprinted gene neuronatin is regulated by metabolic status and associated with obesity.](http://www.ncbi.nlm.nih.gov/pubmed/19851307)*Obesity*(2010) **18(7)**:1289-96. doi: 10.1038/oby.2009.361.

**NNMT (nicotinamide N-methyltransferase)**

**138-** Bromberg A, Lerer E, Udawela M, Scarr E, Dean B, Belmaker RH, et al. [Nicotinamide-N-methyltransferase (NNMT) in schizophrenia: genetic association and decreased frontal cortex mRNA levels.](http://www.ncbi.nlm.nih.gov/pubmed/21791160)*Int J Neuropsychopharmacol*(2012) **15(6)**:727-37. doi: 10.1017/s1461145711001179

**139-** Ulanovskaya OA, ZuhlAM, Cravatt BF.[NNMT promotes epigenetic remodeling in cancer by creating a metabolic methylation sink.](http://www.ncbi.nlm.nih.gov/pubmed/23455543)*Nat ChemBiol*(2013) **9(5)**:300-6. doi: 10.1038/nchembio.1204.

**NR1D1 (nuclear receptor subfamily 1, group D, member 1)**

**140-** Ramakrishnan SN, Muscat GE. [The orphan Rev-erb nuclear receptors: a link between metabolism, circadian rhythm and **inflammation**?](http://www.ncbi.nlm.nih.gov/pubmed/16741567)*NuclRecept Signal*(2006) 4:e009. doi: 10.1621/nrs.04009.

**PDE4DIP (phosphodiesterase 4D interacting protein)**

**141-** DeWan AT, Egan KB, Hellenbrand K, Sorrentino K, Pizzoferrato N, Walsh KM, et al. [Whole-exome sequencing of a pedigree segregating asthma.](http://www.ncbi.nlm.nih.gov/pubmed/23046476)*BMC Med Genet*(2012) **13**:95. doi: 10.1186/1471-2350-13-95.

**PNPLA3 (patatin-like phospholipase domain containing 3)**

**142-** Sookoian S, Pirola CJ. [Meta-analysis of the influence of I148M variant of patatin-like phospholipase domain containing 3 **gene** (**PNPLA3**) on the susceptibility and histological severity of nonalcoholic fatty liver disease.](http://www.ncbi.nlm.nih.gov/pubmed/21381068)*Hepatology*(2011) **53(6)**:1883-94. doi: 10.1002/hep.24283.

**PON3 (paraoxonase 3)**

**143-** Witte I, Foerstermann U, Devarajan A, Reddy ST, Horke S. [Protectors or Traitors: The Roles of PON2 and PON3 in Atherosclerosis and Cancer.](http://www.ncbi.nlm.nih.gov/pubmed/22666600)*J Lipids*(2012) **2012**:342806. doi: 10.1155/2012/342806.

**PTX3 (pentraxin 3, long)**

**144-** Mantovani A, Valentino S, Gentile S, Inforzato A, Bottazzi B, Garlanda C. [The long pentraxin**PTX3**: a paradigm for humoral pattern recognition molecules.](http://www.ncbi.nlm.nih.gov/pubmed/23527487)*Ann N Y AcadSci*(2013) **1285**:1-14.doi: 10.1111/nyas.12043.

**145-** Bastrup-Birk S, Skjoedt MO, Munthe-Fog L, Strom JJ, Ma YJ, Garred P. [Pentraxin-3 serum levels are associated with disease severity and mortality in patients with systemic inflammatory response syndrome.](http://www.ncbi.nlm.nih.gov/pubmed/24039869)*PLoSOne* (2013) **8(9)**:e73119. doi:10.1371/journal.pone.0073119.

**146-** Uusitalo-Seppälä R, Huttunen R, Aittoniemi J, Koskinen P, Leino A, Vahlberg T, RintalaEM.[Pentraxin 3 (PTX3) is associated with severe sepsis and fatal disease in emergency room patients with suspected infection: a prospective cohort study.](http://www.ncbi.nlm.nih.gov/pubmed/23341967)*PLoS One*(2013) **8(1)**:e53661.doi: 10.1371/journal.pone.0053661.

**147-** Kunes P, Holubcova Z, Kolackova M, Krejsek J. [Pentraxin 3(PTX 3): an endogenous modulator of the inflammatory response.](http://www.ncbi.nlm.nih.gov/pubmed/22577258)*Mediators Inflamm*(2012) **2012**:920517. doi: 10.1155/2012/920517.

**RASGRP2 (RAS guanyl releasing protein 2 (calcium and DAG-regulated))**

**148-** Nagamine K, Matsuda A, Hori T. [Identification of the gene regulatory region in human rasgrp2 gene in vascular endothelial cells.](http://www.ncbi.nlm.nih.gov/pubmed/20606303)*Biol Pharm Bull* (2010) **33(7)**:1138-42.doi: 10.1248/bpb.33.1138.

**SARDH (sarcosine dehydrogenase)**

**149-** Khan AP, Rajendiran TM, Ateeq B, Asangani IA, Athanikar JN, Yocum AK, et al. [The role of sarcosine metabolism in prostate cancer progression.](http://www.ncbi.nlm.nih.gov/pubmed/23633921)*Neoplasia* (2013) **15(5)**:491-501.doi: 10.1593/neo.13314.

**SH3D21 (SH3 domain containing 21)**

**150-** Stelzl U, Worm U, Lalowski M, Haenig C, Brembeck FH, Goehler H, et al. [A human protein-protein interaction network: a resource for annotating the proteome.](http://www.ncbi.nlm.nih.gov/pubmed/16169070)*Cell*(2005) **122(6)**:957-68.doi: 10.1016/j.cell.2005.08.029.

**SIGLEC15 (sialic acid binding Ig-like lectin 15)**

**151-**Takamiya R, Ohtsubo K, Takamatsu S, Taniguchi N, Angata T. [The interaction between Siglec-15 and tumor-associated sialyl-Tn antigen enhances TGF-β secretion from monocytes/macrophages through the DAP12-Syk pathway.](http://www.ncbi.nlm.nih.gov/pubmed/23035012)*Glycobiology*(2013) **23(2)**:178-87. doi:10.1093/glycob/cws139.

**152-** Xiong YS, Wu AL, Lin QS, Yu J, Li C, Zhu L, et al. [Contribution of monocytes Siglec-1 in stimulating T cells proliferation and activation in atherosclerosis.](http://www.ncbi.nlm.nih.gov/pubmed/22789514)*Atherosclerosis*(2012) 224(1):58-65. doi: 10.1016/j.atherosclerosis.2012.06.063.

**SKAP1 (src kinase associated phosphoprotein 1)**

**153-** Huang CN, Huang SP, Pao JB, Chang TY, Lan YH, Lu TL, et al. [Genetic polymorphisms in androgen receptor-binding sites predict survival in prostate cancer patients receiving androgen-deprivation therapy.](http://www.ncbi.nlm.nih.gov/pubmed/21652578)*Ann Oncol*(2012) **23(3)**:707-13. doi: 10.1093/annonc/mdr264.

**SLAMF7 (SLAM family member 7)**

**154-** Hsi ED, Steinle R, Balasa B, Szmania S, Draksharapu A, Shum BP, et al. [CS1, a potential new therapeutic antibody target for the treatment of multiple myeloma.](http://www.ncbi.nlm.nih.gov/pubmed/18451245)*Clin Cancer Res*(2008) **14(9)**:2775-84. doi:10.1158/1078-0432.ccr-07-4246.

**SLC15A1 (solute carrier family 15 (oligopeptide transporter), member 1)**

**155-** Ayyadurai S, Charania MA, Xiao B, Viennois E, Merlin D. [PepT1 expressed in immune cells has an important role in promoting the immune response during experimentally induced colitis.](http://www.ncbi.nlm.nih.gov/pubmed/23797361)*Lab Invest*(2013) **93(8)**:888-99. doi: 10.1038/labinvest.2013.77.

**SNX10 (sorting nexin 10)**

**156-** Pangrazio A, Fasth A, Sbardellati A, Orchard PJ, Kasow KA, Raza J, et al. [SNX10 mutations define a subgroup of human autosomal recessive osteopetrosis with variable clinical severity.](http://www.ncbi.nlm.nih.gov/pubmed/23280965)*J Bone Miner Res*(2013) **28(5)**:1041-9. doi: 10.1002/jbmr.1849.

**STAT1 (signal transducer and activator of transcription 1, 91kDa)**

**157-** oromilas AE, Sexl V. [The tumor suppressor function ofSTAT1 in breast cancer.](http://www.ncbi.nlm.nih.gov/pubmed/24058806)*JAKSTAT* (2013) **2(2)**:e23353.doi: 10.4161/jkst.23353.

**158-** Boisson-Dupuis S, Kong XF, Okada S, Cypowyj S, Puel A, Abel L, et al. [Inborn errors of human STAT1: allelic heterogeneity governs the diversity of immunological and infectious phenotypes.](http://www.ncbi.nlm.nih.gov/pubmed/22651901)*CurrOpinImmunol*(2012) **24(4)**:364-78.doi:10.1016/j.coi.2012.04.011.

**TEF (Transcription Enhancer Factors)**

**159-** Jin Y, Messmer-Blust AF, Li J. [The role of transcriptionenhancerfactors in cardiovascular biology.](http://www.ncbi.nlm.nih.gov/pubmed/22498013)*Trends Cardiovasc Med*(2011) **21(1)**:1-5. doi: 10.1016/j.tcm.2011.12.009.

**TMEM91 (transmembrane protein 91)**

**160-** Satoh J, Kawana N, Yamamoto Y. Pathway Analysis of ChIP-Seq-Based NRF1 Target Genes Suggests a Logical Hypothesis of their Involvement in the Pathogenesis of Neurodegenerative Diseases*.Gene RegulSyst Bio*(2013) **7**:139-52.doi: 10.4137/grsb.s13204.

**TNFRSF25 (TNFRSF25 tumor necrosis factor receptor superfamily, member 25)**

**161-** Meylan F, Richard AC, Siegel RM. [TL1A and DR3, a TNF family ligand-receptor pair that promotes lymphocyte costimulation, mucosal hyperplasia, and autoimmune inflammation.](http://www.ncbi.nlm.nih.gov/pubmed/22017439)*Immunol Rev*(2011) **244(1)**:188-96. doi: 10.1111/j.1600-065x.2011.01068.x.

**TOX2 (TOX high mobility group box family member 2)**

**162-**Tessema M, Yingling CM, Grimes MJ, Thomas CL, Liu Y, Leng S,et al. [Differential epigenetic regulation of TOX subfamily high mobility group box genes in lung and breast cancers.](http://www.ncbi.nlm.nih.gov/pubmed/22496870)*PLoS One*(2012) **7(4)**:e34850. doi: 10.1371/journal.pone.0034850.

**UBD (ubiquitin D)**

**163-** Yuan J, Tu Y, Mao X, He S, Wang L, Fu G, et al. [Increased expression of FAT10 is correlated with progression and prognosis of human glioma.](http://www.ncbi.nlm.nih.gov/pubmed/22402871)*PatholOncol Res*(2012) **18(4)**:833-9*.*doi: 10.1007/s12253-012-9511-2.

**164-** Chen J, Chen ZJ. [Regulation of NF-κB by ubiquitination.](http://www.ncbi.nlm.nih.gov/pubmed/23312890)*CurrOpinImmunol*(2013) **25(1)**:4-12. doi: 10.1016/j.coi.2012.12.005.

**165-** Edelmann MJ, Nicholson B, Kessler BM. Pharmacological targets in the **ubiquitin** system offer new ways of treating cancer, neurodegenerative disorders and infectious diseases. *Expert Rev Mol Med*(2011) **13**:e35. doi: 10.1017/s1462399411002031.

**166-** Molineaux SM. [Molecular pathways: targeting proteasomal protein degradation in cancer.](http://www.ncbi.nlm.nih.gov/pubmed/22019514)*Clin Cancer Res*(2012) **18(1)**:15-20. doi: 10.1158/1078-0432.ccr-11-0853.

**167-** Napetschnig J, Wu H. [Molecular basis of NF-κB signaling.](http://www.ncbi.nlm.nih.gov/pubmed/23495970)*Annu Rev Biophys*(2013) **42**:443-68.doi: 10.1146/annurev-biophys-083012-130338.
